# High-resolution in situ imaging reveals size-specific moonlight responses in zooplankton diel vertical migration

**DOI:** 10.1038/s41598-026-36105-0

**Published:** 2026-01-28

**Authors:** Ashton L. Dickerson, Andreas Jechow, Michelle Nößler, Tim J. W. Walles, Stella A. Berger, Franz Hölker, Jens C. Nejstgaard

**Affiliations:** 1 Department of Community and Ecosystem Ecology, Leibniz Institute of Freshwater Ecology and Inland Fisheries (IGB), 12587 Berlin, Germany; 2 Brandenburg University of Applied Sciences, 14770 Brandenburg a.d.H, Germany; 3 Department of Plankton and Microbial Ecology, Leibniz Institute of Freshwater Ecology and Inland Fisheries (IGB), Zur alten Fischerhütte 2, 16775 Stechlin, Germany; 4 Institute of Biology, Freie Universität Berlin, 14195 Berlin, Germany

**Keywords:** Ecology, Ecology, Zoology

## Abstract

Light is the primary cue driving zooplankton diel vertical migration (DVM), a strategy that balances predation risk with resource access. However, DVM is often oversimplified, with limited consideration of how light-driven risks and resource needs vary across taxa and life stages. This simplification is partly due to constraints on collecting high-resolution, size-resolved data —especially at night, when subtle shifts in illumination reshape nocturnal risk landscapes. To overcome these limitations, we deployed a high-resolution in situ modular Deep-focus Plankton Imager and an image-recognition approach to quantify fine scale DVM and body sizes of Cladocerans and Copepods in Lake Stechlin, Germany. Data was collected from day into night and across moonrise and was compared with environmental data from vertical profiling sondes. Typical DVM patterns emerged, with deeper daytime distributions, however, moonlight introduced additional behavioural complexity: larger individuals avoided illuminated layers, likely managing predation risk, while smaller individuals moved into these layers, possibly exploiting foraging opportunities and reduced risk. These light-mediated shifts were further shaped by ecological conditions; copepods tracked food-rich layers regardless of light levels at night, while cladocerans showed light-dependent responses to both temperature and food, such that light caused them to avoid otherwise favourable (warm, food-rich) layers. Our approach provides new insight into how zooplankton navigate nocturnal lightscapes, revealing size- and taxon-specific strategies. By establishing size-dependent responses to natural moonlight, this work provides a crucial baseline for predicting how artificial light at night may restructure zooplankton communities and destabilize freshwater food webs.

**Supplementary Information:**

The online version contains supplementary material available at 10.1038/s41598-026-36105-0.

## Introduction

Diel vertical migration (DVM) of zooplankton is the largest synchronized migration on Earth, a behaviour studied for over two centuries^1,2^. Light is widely recognized as the primary proximate factor regulating DVM timing and magnitude, with zooplankton migrating downward during the day to avoid visual predators and harmful UV radiation, then ascending at night to access warmer, productive surface waters^3–6^. In addition to the day-night cycle, subtle nocturnal light variations, such as changes in moonlight intensity, also influence DVM by altering predation risk landscapes^3,7,8^. However, much DVM research oversimplifies zooplankton responses by treating light as the sole driver, neglecting the complex trade-offs individuals navigate. In reality, DVM is shaped by the interplay of light-induced predation risk, prey availability, and environmental conditions^9,10^, yet these have rarely been examined in combination in the field. This gap partly reflects historical limitations in observing zooplankton movements with sufficient temporal, spatial, and size resolution to capture fine-scale responses in situ. Further, while large marine zooplankton such as krill undertake extensive migrations that can be tracked acoustically, e.g.^11,12^, freshwater zooplankton are dominated by smaller taxa such as *Daphnia* and copepods, whose vertical migrations span only a few metres e.g.^13,14^, and references therein. These fine-scale movements are difficult to detect with traditional net sampling due to limited temporal and depth resolution, compounded by environmental variability such as weak thermal stratification and shallow depths 1–4. Thus, the lack of methods to adequately document migration of small-bodied freshwater zooplankton has limited understanding of their behavioural dynamics^15^, and specifically calls for new high-resolution approaches^1^.

Zooplankton predation risk under varying light levels depends on body size and escape performance, which in turn vary with species, developmental stage, and physiological state. Smaller individuals, such as early developmental stages or smaller species, are less detectable by predators and have less swimming capabilities, and may therefore remain at depths with optimal resources regardless of light^8,16–19^. In contrast, larger zooplankton (e.g., larger species and developmental stages, egg-carrying females) are more visible to predators, but can also benefit from faster swimming speeds that enhance their escape capabilities, as seen in larger copepods^19,20^. Further, physiological light sensitivity increases with development, as photoreceptors mature and increase in complexity through successive life stages^21,22^. These size, species and ontogenetic related differences lead to varied migration patterns, where larger zooplankton exhibit more pronounced vertical movements in response to light. Additionally, light-induced risk only becomes significant when required resources or conditions occur in brighter (i.e. shallower) water layers. Since resource needs and optimal conditions differ across life stages, motivation to migrate varies accordingly. For example, young zooplankton seek warmer temperatures, which enhance growth and shorten generation times; these temperatures are typically found near (brighter) surfaces^23^. Conversely, mature females may prioritise food to increase egg clutch size^24,25^, though the depth of the food maxima varies across ecosystems^26^. Motivation also fluctuate dynamically with internal state; hunger or warmer temperatures may raise an individual’s tolerance to light, delaying downward migration despite higher predation risk^27^. Thus, vertical migration responses to light-driven predation pressure reflect a complex interplay of individual traits, intrinsic motivation, and ecosystem factors.

Our limited understanding of fine-scale DVM patterns partly stems from historical data-collection constraints, particularly for mesozooplankton^1^. Early net-tow methods provide only coarse estimates across metre-scale depth strata, whereas freshwater zooplankton interact with prey and predators over millimetres to centimetres and on minute-to-second timescales. Manual, labour-intensive net and microscope approaches therefore lack the spatial and temporal resolution required to capture such fine-scale behaviours. Acoustic sampling improves spatial resolution and, with broadband acoustics, can differentiate zooplankton groups across size classes based on spectral backscatter^28^, but cannot reliably identify individual taxa or measure biological traits such as body size or developmental stage. Underwater imaging, particularly the modular Deep-focus Plankton Imager (mDPI), provides images with a practical resolution of 30–60 μm, allowing accurate identification of crustacean zooplankton larger than ca. 300–400 μm across the whole 10 cm depth of field (see Fig. 8 in Greer et al. 2025). This relatively high practical resolution is critical as most crustacean that dominate freshwater systems, like Lake Stechlin^13,^ are smaller than ~ 1 mm. The mDPI also enables automated acquisition of large datasets and, when combined with automated image recognition algorithms, facilitates detailed studies of DVM by specific taxa and size classes^1,29–31^. These technological advances now allow unprecedented insights into how subtle light variations, including moonlight, shape zooplankton vertical migration.We used a mDPI to profile zooplankton vertical distribution in Lake Stechlin, Germany, a clear, deep, temperate lake with an abundant zooplankton community and high densities of planktivorous fish^32,33^. We conducted high-resolution sampling of zooplankton across diel periods during both new- and full-moon phases, alongside measurements of temperature and chlorophyll-*a* (as a proxy for food availability). Our aim was to assess how light influences DVM, with a particular focus on size-specific behavioural responses. We expected to observe classic DVM patterns, with zooplankton migrating to deeper water layers during the day to avoid predators and harmful UV exposure, and ascending at night to exploit warmer, food-rich surface waters. We further hypothesised that responses to nocturnal light would vary with body size. Smaller zooplankton, being less detectable to visual predators, were expected to show limited migration and instead remain in zones with favourable food and temperature conditions. In contrast, we expected that larger zooplankton would exhibit more pronounced DVM, retreating to deeper water layers during moonlit nights to reduce light-mediated predation risk. By examining these size-specific vertical migration behaviours, our study provides new insight into the trade-offs underlying DVM under varying nocturnal light conditions.

## Methods

### Study site and organisms

We quantified zooplankton DVM in Lake Stechlin (53°10′N, 13°02′E), a di-monomictic, meso-eutrophic hardwater lake in northeastern Germany^32–34^ with virtually no light pollution^35^. Previous research has documented a large community of planktivorous fish in Lake Stechlin^32,36–38^. For this field study, we utilized the permanently installed large-scale experimental facility in the south bay of Lake Stechlin, the LakeLab, which provided the necessary infrastructure for our data collection, including electricity for the mDPI (modular Deep-focus Plankton Imager, an underwater imaging system for profiling zooplankton distributions) and a monitoring station that records hourly vertical profiles of light (photosynthetic active radiation, PAR) and environmental variables (temperature, oxygen, chlorophyll-*a*, phycocyanin, phycoerythrin). Profiles were taken from the southern side of the LakeLab in the open lake (53°08’34.7"N 13°01’41.1"E, Supplementary Fig. S1) to the full depth of 16.7 m. We focused on mesozooplankton, including cladocerans and copepods, as these taxa are abundant at this site^32,33^, known to migrate^39^, and of a size suitable for image-recognition analysis (details below).

### Data collection schedule

Our field campaign was conducted in late summer to early autumn in 2022 across two lunar cycles, targeting nights close to new moons (25 Aug, 3.8% illumination; 29 Sep, 15% illumination) and full moons (13 Sep, 91.5% illumination; 12 Oct, 94.5% illumination). Sampling was planned for nights with minimal cloud cover to ensure consistent light conditions, avoiding both the masking of moonlight and potential cloud-enhanced amplification of artificial light. For new moon phases, we ensured the moon was below the horizon before sunset and remained so during sampling. For full moon phases, sampling occurred during waning gibbous phases that rose after sunset, allowing us to capture this transitional phase of light (i.e. moonrise). Zooplankton distributions were profiled once during daylight (at ~ 16:00 CET), followed by half-hourly profiles starting 30 min before sunset, continuing for five hours (new moons) or until the moon reached an altitude of at least 50° (full moons). This resulted in 5 to 5.5 h of night sampling per night (mean profiles ± SE = 17.5 ± 1; total = 70 profiles). All equipment was set up during the day, no artificial lights were used during sampling, and we remained in darkness between profiles. When necessary, we occasionally used dim red headlamps to ensure minimal disturbance to natural light conditions.

### Sampling methodology and environmental variables

In situ sampling of mesozooplankton vertical distribution were collected using the mDPI (Bellamare, LLC, San Diego, CA, USA; Greer et al., 2025). The mDPI comprises of a camera pod mounted opposite to a light source pod, each fitted with identical collimator lenses 10 cm apart (Supplementary Fig. S2; Greer et al., 2025). The light source projects parallel rays, while the camera records shadow images of particles at a theoretical optical resolution of 21 μm per pixel, which is numerically derived from the measured image field compared to the pixel count of the sensor. However, the measured practical resolution between the two pods is ca. 30–60 μm for most of the light path (see Fig. 8 in Greer et al. 2025). This results from inevitable optical imperfections in the lens system and distortion of the light rays when passing the 10 cm water path. In order to not overstate the accuracy of the instrument and to make sure the organisms are correctly determined, we therefore only include organisms with an equivalent spherical diameter (ESD, representing the diameter of a sphere enclosing the zooplankton’s volume, calculated from measured pixel size) > 0.36 mm ESD, i.e. longer than ca. 400 μm. Thus, we only report the behaviour of late development stages for copepods and mostly adult cladocerans. The total biomass of the relatively large cladocerans detected in the mDPI (ESD 0.36–0.75 mm) was dominated by *Daphnia cucullata* 47–81% and *Diaphanosoma brachyurum* 19–33%, while the total biomass of the relatively large copepods (ESD 0.36–0.68 mm) was dominated by *Eudiaptomus gracilis* 62–65% and *Eurytemora lacustris* 10–17%, in Aug and Sep 2022. The copepod biomass data used here was obtained by microscopy samples from the Lake Stechlin long term monitoring (J. Nejstgaard and C. Dilewski unpubl.), and does not include the nauplii biomass as they are not included in the detected zooplankton.

The light pod is fitted with a near infrared light source (> 750 nm, light-emitting diode, LED) to minimize potential influence of light on the escape response or behaviour of organisms (e.g. Cohen & Forward^21^). This shadowgraph technique ensures that organisms maintain their original size and proportion regardless of position. The mDPI records 2.4 images s^−1^ at an image size 43 mm high x 52 mm broad. A high-performance depth sensor monitors the lowering speed through the water column. We manually lowered the mDPI using a hand-operated winch, accompanied by a metronome to keep a steady pace, at a target speed ~ 5 m min^−1^ resulting in each profile taking approximately 4 min to collect. Each full vertical measurement, referred to as a “profile”, comprises a complete set of images, with overlapping portions of consecutive images removed based on precise imaging depth. For each mDPI profile, we recorded lunar phase and altitude using information from www.timeanddate.com, along with in-person observations of cloud cover in oktas (the proportion of the sky covered by clouds in eighths).

Water temperature, dissolved oxygen, chlorophyll-*a*, phycocyanin and phycoerythrin concentration, as well as PAR were recorded hourly at 50 cm intervals down to a depth of 16.5 m using the LakeLab profiler system equipped with YSI EXO2-100 multiparameter sondes (water temperature EXO599870, oxygen concentration EXO599100-01, chlorophyll-*a* EXO599102-01, phycoerythrin EXO599103-01; YSI, Yellow Springs, USA) and light sensors (underwater spherical sensor LI-193SA; LI-COR Environmental, Lincoln, USA). As zooplankton can perceive very low light levels^22,40^, we calculated light underwater to obtain finer-scale data than could be provided by the light (PAR) sensors, which are primarily designed to measure daylight irradiance. Illuminance, measured in lux (lx; a measure of light intensity relevant to visual perception), in 10 cm depth bins were estimated using surface illuminance modelled with the R package *moonlit*^41^, accounting for lunar phase, elevation, brightness, and atmospheric effects. We then applied light attenuation calculations to estimate light through the water column (see Supplementary Information, *Underwater illuminance calculations*). Illuminance values below 0.001 lx were treated as zero, representing the lowest scotopic visual sensitivity recorded in fish species^42,43^. For orientation, 0.001 lx corresponds to approximately 0.9–6.5 × 10^− 5^ µmol m^−2^ s^−1^ and 2.1–16.1 × 10^− 6^ W m^−2^ assuming blue–green underwater spectra (monochromatic approximation at 520–480 nm).

### Data processing

We used our image processing pipeline specifically developed for the mDPI. It includes several steps to automatically detect, classify, and count zooplankton in vertical imagery profiles. First, raw images are labelled with metadata, including sampling date, location, and depth. Image segmentation is performed post-acquisition using custom scripts implemented in Python (v3.5). This process binarizes the raw images to separate particles from the background and extracts individual regions of interest (ROIs), each containing a single zooplankton or particle^31^. Following segmentation, the images are then flat-fielded to reduce optical variations, a process that corrects for uneven illumination and sensor sensitivity across the image field. ROIs containing zooplankton are identified and cropped into vignettes, with size thresholds set to exclude objects that are either too small or too large. These vignettes are then used as input for a Convolutional Neural Network (CNN^44^) implemented in Keras (https://keras.io) with TensorFlow (https://www.tensorflow.org) to classify mesozooplankton into taxonomic classes. Here we focused on the broad groups cladocerans or copepods, which are representative of common taxa found in freshwater environments^14^.

The CNN was pre-trained using an active learning framework that minimized manual annotation effort by iteratively selecting the most informative samples for labelling, while pseudo-labelling high-confidence samples to enrich training data. The classifier architecture is based on AlexNet^45^, adapted for plankton image data, and trained with dropout and cross-entropy loss to prevent overfitting and optimize classification. Model performance was evaluated on multiple datasets, achieving baseline accuracy of 83.8%, improving to 96% accuracy after fine-tuning on additional data^31^. Prediction confidence scores (softmax probabilities) were used as quality control during training to select samples for manual annotation or pseudo-labelling.

To classify zooplankton into size categories (small, medium, and large), we used the ESD. The ESD values were converted into millimetres using a pixel size of 21 μm (divided by 1000) and were subsequently used to categorise the size histogram into three distinct size classes for cladocerans and copepods separately. For cladocerans, small individuals ranged from 0.36 to 0.39 mm, medium from 0.45 to 0.53 mm, and large from 0.70 to 0.75 mm. For copepods, small individuals ranged from 0.36 to 0.37 mm, medium from 0.45 to 0.47 mm, and large from 0.66 to 0.68 mm (Supplementary Table S1). Density (ind*L^−1^) of each size class for both taxa was calculated within 10 cm vertical bins throughout the water column, resulting in 167 bins per profile. The density of zooplankton within each bin was used for analyses. This 10 cm bin resolution balances small-scale vertical resolution while still maintaining a meaningful number of zooplankton within each bin.

### Data analysis and statistics

#### Summary visualisation

To visualise diel patterns in zooplankton vertical distribution across time, we calculated the average depth (± SE) of each taxonomic group (cladocerans and copepods) per profile. For this, we used the actual depth of each identified zooplankton individual (not binned densities), and computed the mean and standard error of individual depths per group per profile. SE was chosen over standard deviation (SD) to better visualise overall trends, as SD values were large and obscured the underlying patterns. These values were used for the time-series summary figure only and were not included in statistical models.

#### Overall effect of light across the day–night transition

To test how zooplankton density (ind*L^−1^) across depth bins responded to changing light conditions from daytime into night-time, we used generalized linear mixed models (GLMMs) with a gamma distribution and log link function (chosen to account for positive, continuous, right-skewed data) (R version 4.2.2, package lmerTest^46^). Separate models were constructed for cladocerans and copepods. In each model, zooplankton concentration within each bin was the dependent variable, with illuminance (lx) and size class (small/medium/large) as independent factors. We included an interaction between illuminance and size class, as we hypothesize that light effects would vary by size. Sampling date and profile ID were included as random factors to account for seasonal variation and multiple observations within profiles. Cloud cover after sunset was minimal (mean oktas = 0.87; range = 0–3), so it was excluded from the models.

#### Effect of changes of light at night

We examined how light at night (twilight and moonlight) influenced zooplankton vertical positioning and whether this response was mediated by environmental factors. For this, we subset the data to include only profiles taken after civil twilight (the period just after sunset and before sunrise when the sun is 0–6° below the horizon and some natural light is still present). Illuminance values after civil twilight ranged from 0.00 to 0.06 lx (underwater). For orientation, assuming a blue–green underwater spectrum (500 nm), this corresponds to ~ 0–1.14 × 10^−3^ µmol m^− 2^ s^−1^ and ~ 0–2.72 × 10^− 4^ W m^− 2^. Separate models were again constructed for cladocerans and copepods, with zooplankton density (ind*L^−1^) across depth bins as the dependent variable. The independent factors were illuminance (lx), size class (small/medium/large), temperature, and chlorophyll-*a* concentration at each depth bin. We specifically tested two three-way interactions: illuminance × size class × temperature and illuminance × size class × chlorophyll-*a* concentration. These interactions were included to assess how zooplankton of different sizes respond to varying light conditions alongside environmental factors, as they experience different levels of light-induced predation risk based on size and have distinct resource requirements. Sampling date and profile ID were included as random factors. As underwater illuminance varies continuously across new- and full-moon periods (e.g. due to lunar altitude), we modelled illuminance as a continuous predictor rather than categorising nights by moon phase. This structure ensures that any differences associated with new- versus full-moon sampling periods are appropriately captured within the random effect of sampling date, while allowing the model to quantify responses to the actual light levels experienced by zooplankton.

#### Statistical inference

For all models, we calculated 95% highest posterior density (HPD) confidence intervals (CI) for each fixed effect using parametric bootstrap simulations with 10,000 iterations and considered CIs that did not overlap zero to have a significant effect^47–49^. We also estimated variance components for random effects with 95% HPD intervals. All models converged successfully, and residual diagnostics confirmed model assumptions were met.

## Results

### Environmental variables

**Fig. 1:**
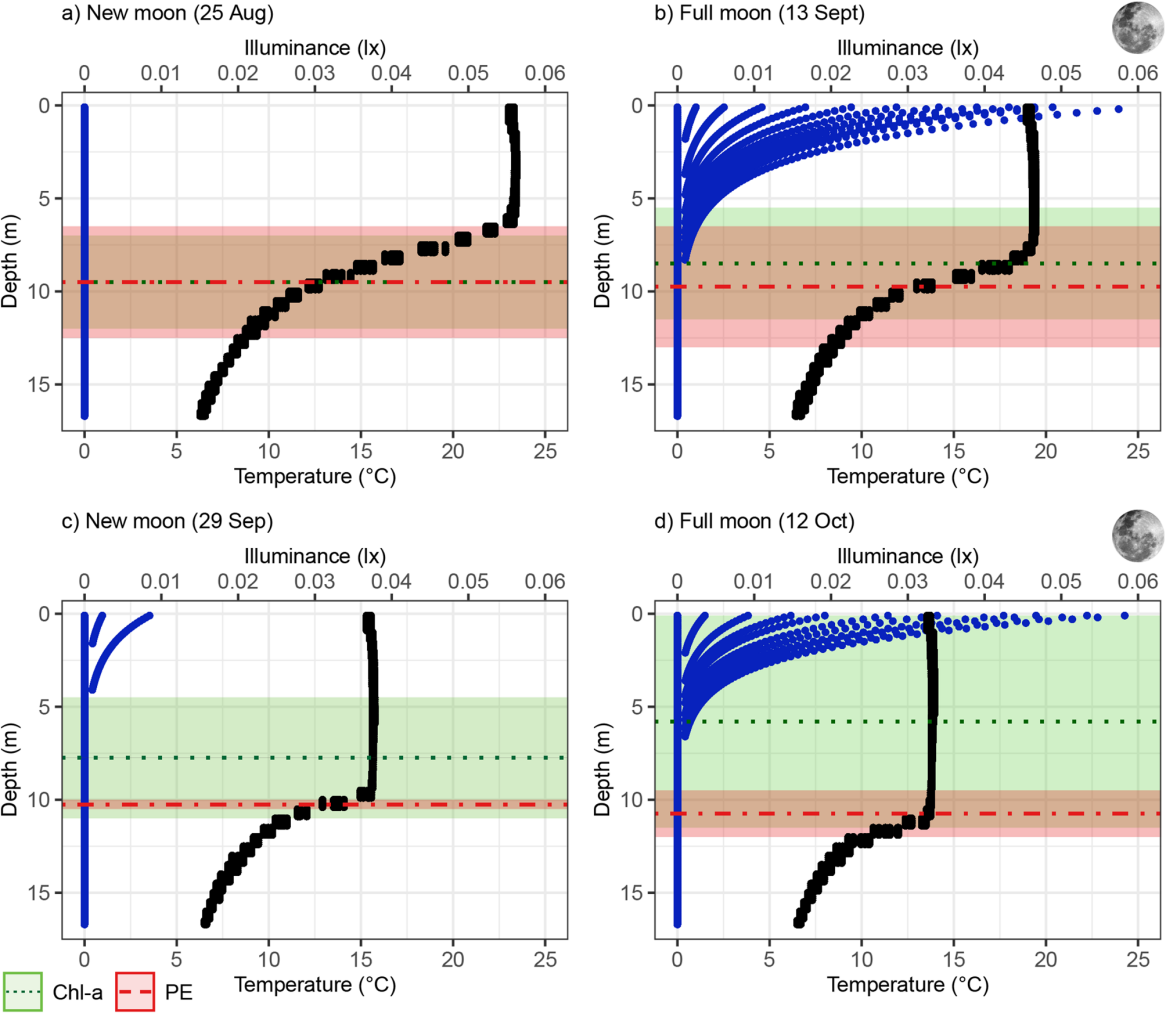
Distribution of environmental variables measured during the night (including nautical twilight, astronomical twilight, and true night) against depth at Lake Stechlin. Black points represent temperature, while blue points indicate calculated lx values at 50 cm intervals. For both chlorophyll-*a* (dark green) and phycoerythrin (PE; red), horizontal lines show the mean depth at which each pigment reached its maximum concentration across profiles, and the corresponding shaded ribbons indicate the ranges of these maximum-depth values.

Chlorophyll-*a* concentrations were relatively consistent through the water layers across recording nights (Supporting Information Fig. S3), but peaked at deeper depths (Fig. 1). The use of chlorophyll-*a* as a food proxy in this system was supported by low phycocyanin levels (a proxy for cyanobacteria) and deeper peaks in phycoerythrin (a proxy for toxic cyanobacteria consisting mainly of *Planktothrix rubescens*) compared to chlorophyll-*a* (Supporting Information Fig. S3), suggesting chlorophyll-*a* levels accurately represented viable food sources. Oxygen levels remained consistently above 3 mg L^−1^ throughout the water column across recording nights (Supporting Information Fig. S3), indicating no hypoxic conditions relevant to zooplankton migration^50^. Additionally, illuminance values above 0.001 lx during the night were restricted to the epilimnion (Fig. 1).

### Overall effect of light (day-night)

**Fig. 2:**
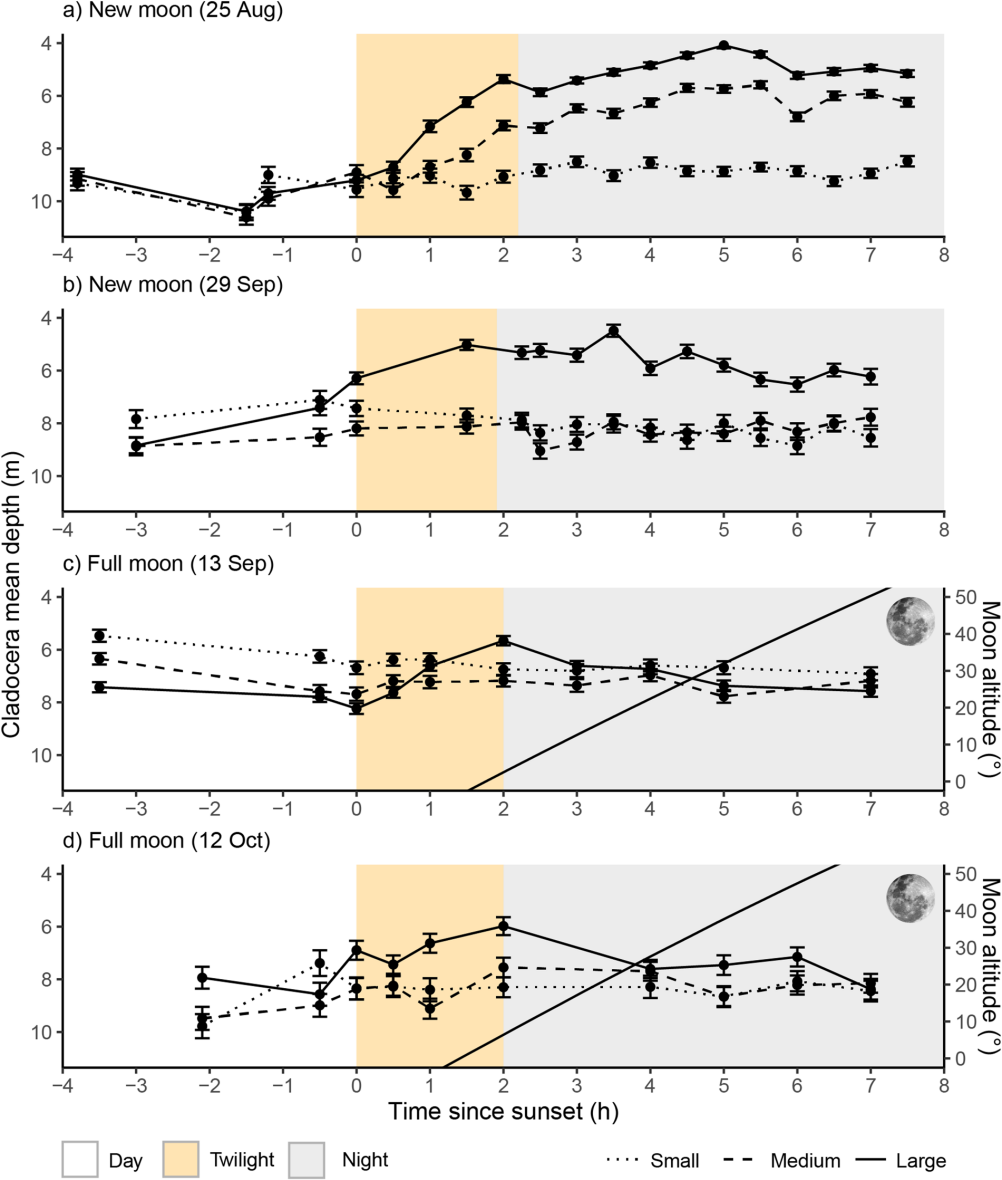
Mean and standard error of the average depth (m) of small, medium and large cladocerans across time since sunset (h) during, new- (a and b) and full moons (c and d) measured in Lake Stechlin, Germany (N 10 cm bin depths sampled per data point = 167). Solid line in graphs c and d represents the moon altitude. Yellow represents nautical twilight to astronomical twilight and grey represents true night. Technical difficulties during new moon sampling of 29/09/2022 meant two profiles were incomplete and therefore not used to calculate mean depths (30 min and 1 h after sunset).

**Fig. 3:**
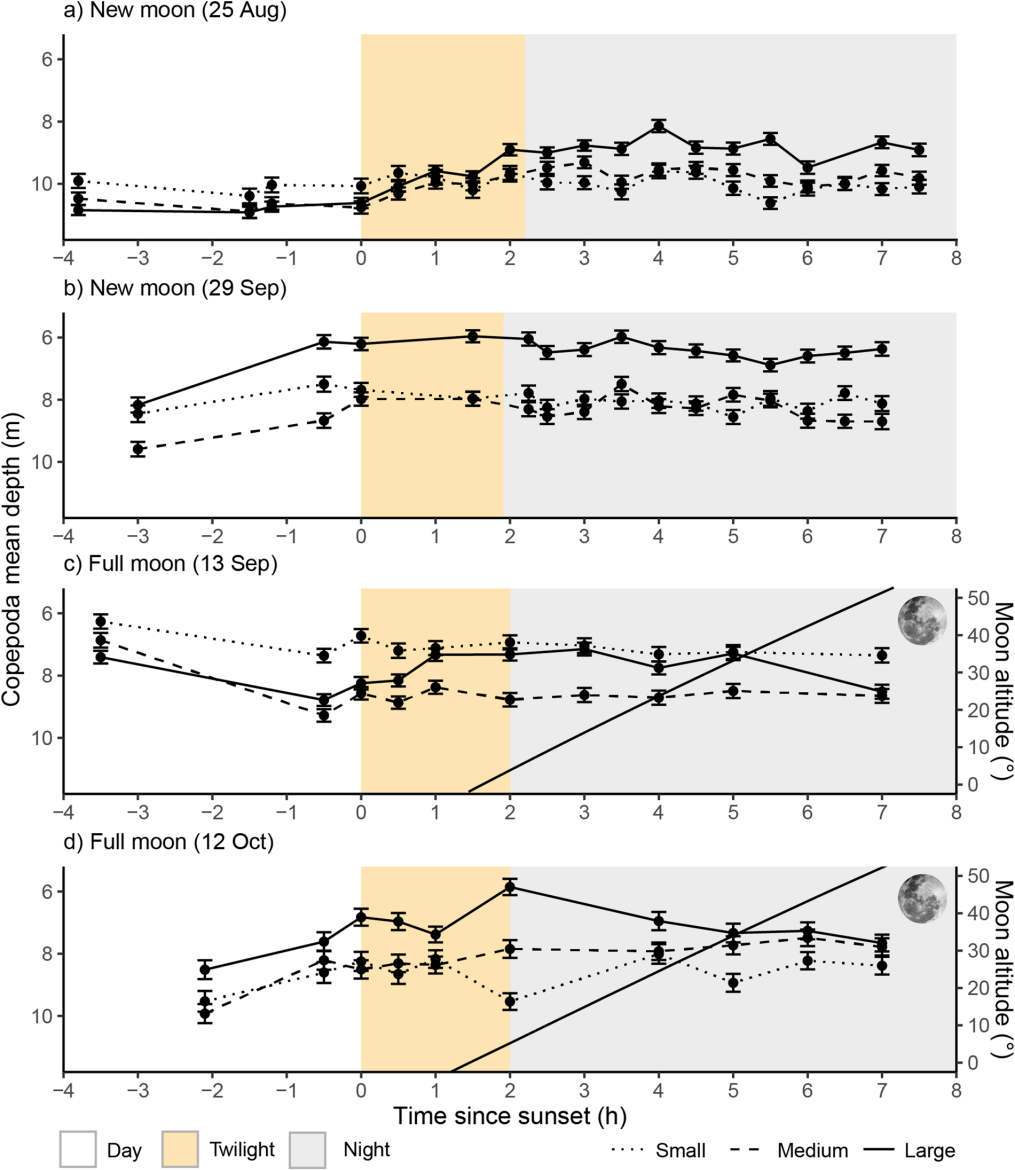
Mean and standard error of the average depth (m) of small, medium and large copepods across time since sunset (h) during, new- (a and b) and full moons (c and d) measured in Lake Stechlin, Germany (N 10 cm bin depths sampled per data point = 167). Solid line in graphs c and d represents the moon altitude. Yellow represents nautical twilight to astronomical twilight and grey represents true night. Technical difficulties during new moon sampling of 29/09/2022 meant two profiles were incomplete and therefore not used to calculate mean depths (30 min and 1 h after sunset).

Zooplankton showed clear vertical distribution shifts between day and night, with both cladocerans and copepods occurring at shallower water layers at night than during the day (Figs. 2 and 3). These patterns were particularly pronounced in larger individuals of both species’ groups, which exhibited stronger DVM than medium or small-sized individuals (see Video S1-S8 for animated depth profile change over time for each size group). Notably, large zooplankton adjusted to a deeper position in line with rising full moons (Figs. 2 and 3). Across cladocerans and copepods, daytime depths were greater than night depths. During new-moon nights, cladocerans had a mean daytime depth of 9.13 m (SD = 4.24 m) and night depth of 6.93 m (SD = 4.25 m), while copepods had a mean daytime depth of 9.83 m (SD = 3.96 m) and night depth of 8.62 m (SD = 4.21 m). During full-moon nights, cladocerans had a mean daytime depth of 7.47 m (SD = 4.33 m) and night depth of 6.92 m (SD = 4.40 m), while copepods had a mean daytime depth of 8.08 m (SD = 4.25 m) and night depth of 7.54 m (SD = 4.52 m) (Figs. 2 and 3).

**Fig. 4:**
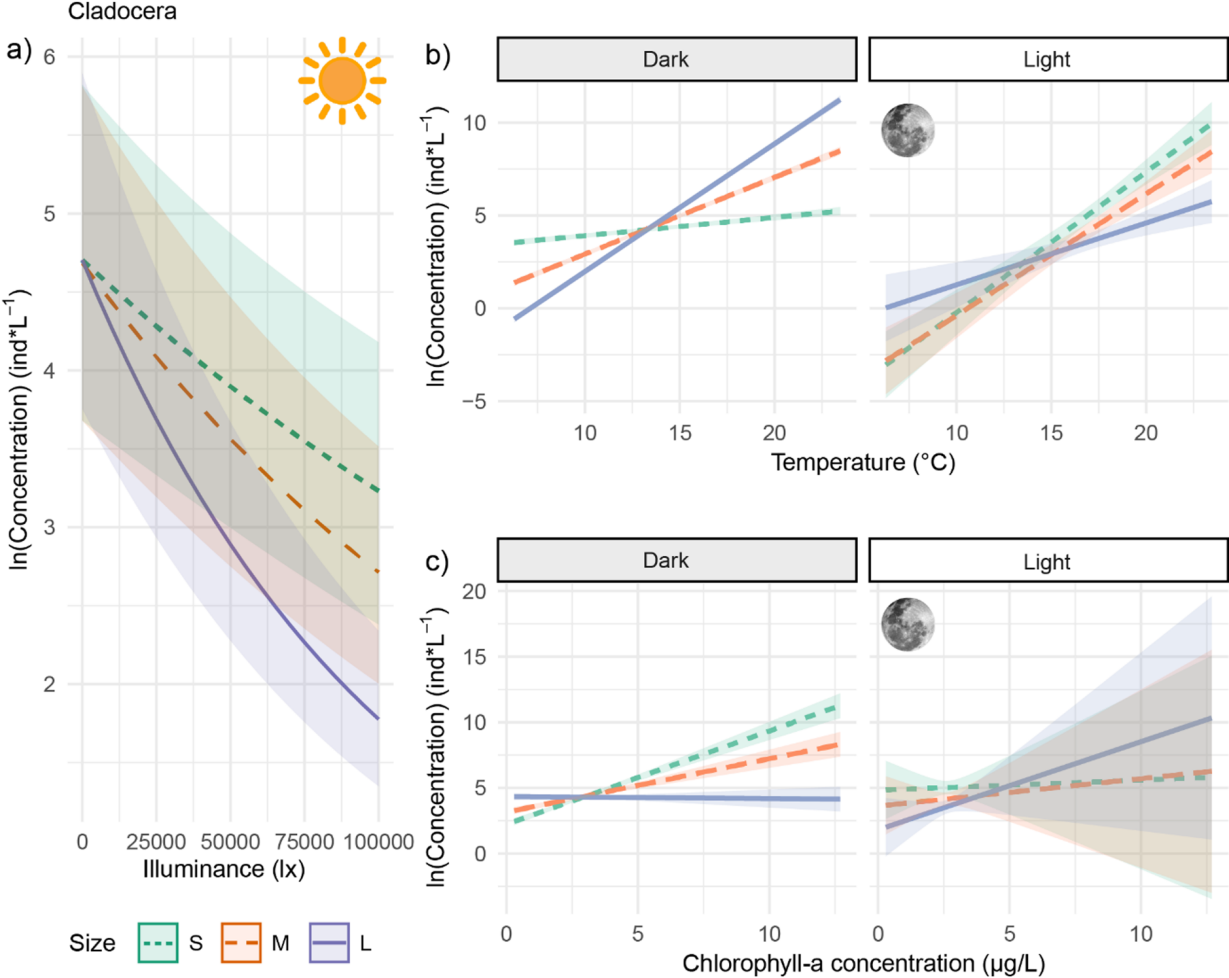
Concentration (log-transformed) of small, medium, and large cladocerans across 10 cm depth bins in relation to calculated illuminance (lx) from day to night (panel a; *N* = 35,070 samples per graph). Panels b and c show predicted concentrations in relation to temperature (°C) and chlorophyll-*a* (µg l^−1^), respectively, under dark conditions (illuminance < 0.001 lx) or light conditions (illuminance 0.01–0.06 lx) at night, including nautical and astronomical twilight (*N* = 27,054 depth bins sampled). Data were collected in Lake Stechlin, Germany, across two lunar cycles. Light is separated categorically for illustration purposes but was included as a continuous variable in the statistical models. Trend lines shows the model-predicted effects, ribbons shows the standard error of this estimate.

**Fig. 5:**
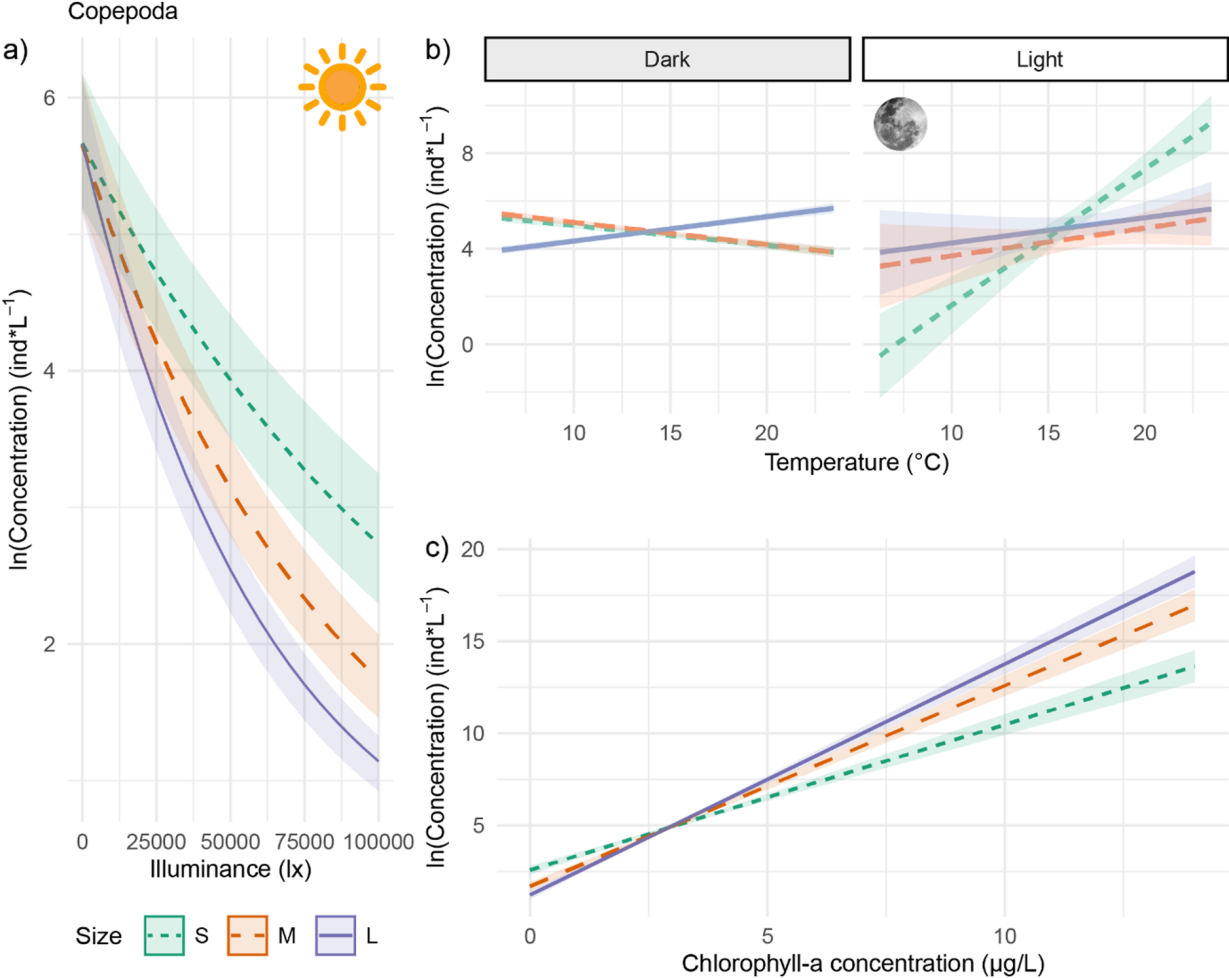
Concentration (log-transformed) of small, medium, and large copepods across 10 cm depth bins in relation to calculated illuminance (lx) from day to night (panel a; *N* = 35,070 samples per graph). Panels b and c show predicted concentrations in relation to temperature (°C) and chlorophyll-*a* (µg l^−1^), respectively, under dark conditions (illuminance < 0.001 lx) or light conditions (illuminance 0.01–0.06 lx) at night, including nautical and astronomical twilight (*N* = 27,054 depth bins sampled). Light is shown categorically for illustration, but was included as a continuous variable in the statistical models. Trend lines indicate model-predicted effects, with ribbons representing standard errors. Data were collected in Lake Stechlin, Germany, across two lunar cycles. Light is separated categorically for illustration purposes but was included as a continuous variable in the statistical models. Trend lines shows the model-predicted effects, ribbons shows the standard error of this estimate.

The GLMMs models examining the overall effect of light showed that zooplankton density was lower in water layers with higher illuminance, indicating that both cladocerans and copepods avoided brighter strata of the water column (Table 1; Figs. 4 and 5). Among cladocerans, small and medium individuals exhibited a similar decline in density with increasing light, while large individuals avoided illuminated water layers even more strongly (Table 1; Fig. 4a). For copepods, avoidance of brighter conditions became progressively more pronounced with increasing size groups (Table 1; Fig. 5a).

**Table 1:** The effect of light (across day to night, lx) and size class (small, medium, large) on density of cladocerans and copepods measured in lake Stechlin, Germany, across two lunar cycles (N 10 cm bins sampled = 35,070). Estimated effect sizes are shown for fixed effects with 95% confidence intervals and random effects show the variance attributed to date (*N* = 4) and profiles (*N* = 70) as calculated with the bootstrap method (10,000 iterations). Bold text represents significant effects.

	Cladocera	Copepoda
Fixed effects	β (95% CI)	β (95% CI)
Intercept	1.55 (1.26, 1.83)	1.75 (1.61, 1.85)
Light (day to night, lx)	**-3.98**^**− 6**^ **(-5.89**^**− 6**^, **-1.81**^**− 6**^**)**	**-7.42**^**− 6**^ **(-9.24**^**− 6**^, **-5.27**^**− 6**^**)**
Size class (compared to small)		
*Medium*	-3.31^−3^ (-3.05^− 2^, 2.14^− 2^)	-3.90^−3^ (-2.78^− 2^, 2.38^− 2^)
*Large*	-3.74^−3^ (-2.64^− 2^, 2.65^− 2^)	-1.70^−3^ (-2.56^− 2^, 2.57^− 2^)
Lx : size class (*medium*)	-1.80^− 6^ (-4.38^− 6^, 1.17^− 6^)	**-4.50**^**− 6**^ **(-7.31**^**− 6**^, **-1.88**^**− 6**^**)**
Lx : size class (*large*)	**-5.52**^**− 6**^ **(-8.75**^**− 6**^, **-3.35**^**− 6**^**)**	**-8.61**^**− 6**^ **(-1.15**^**− 5**^, **-6.14**^**− 6**^**)**
**Random effects**	**σ2 (95% CI)**	**σ2 (95% CI)**
Date	0.11 (0.03, 0.22)	0.01 (0.00, 0.04)
Profile	0.03 (0.03, 0.04)	0.01 (0.01, 0.01)

### Effect of changes in light at night

After civil twilight (i.e. during nautical twilight, (sun 6–12° below the horizon*)*; astronomical twilight, (sun 12–18° below the horizon); and night), underwater illuminance ranged from 0.00 to 0.06 lx. The GLMMs indicated that zooplankton density varied in response to illumination, temperature, and chlorophyll-*a* concentration, but the strength and direction of these responses depended on size (Table 2; Figs. 4 and 5).

### Cladocera

#### Temperature

Cladoceran density increased with warmer water, but the magnitude of this response varied across size classes and illumination levels (Table 2; Fig. 4b). Under low-light conditions, larger individuals became more concentrated in warmer water, while medium-sized individuals showed the same but weaker trend. Small individuals exhibited minimal change (Fig. 4b). However, under brighter nighttime conditions, the pattern was reversed: small individuals showed the strongest increase in density with rising temperatures, followed by medium-sized individuals, with large individuals being least affected (Fig. 4b).

#### Food availability

In darkness, the density of small and medium cladocerans increased with higher chlorophyll-*a* concentrations, with the strongest response observed in small individuals (Table 2; Fig. 4c). Large individuals, however, did not show a relationship with chlorophyll-*a*. Under illuminated conditions, cladoceran responses to food availability were highly variable across size classes. A significant interaction between light and chlorophyll-*a* for large individuals (GLMM, β = 0.07, 95% CI [0.00, 0.13]) suggests that they were found at higher densities in food-rich water layers only when light was present (Table 2; Fig. 4c).

**Table 2:** The effect of light (twilight to moonlight, lx), temperature (temp), chlorophyll-*a* (Chl *a*) concentration and size class (small, medium, large) on concentration of cladocerans and copepods as measured in lake Stechlin, Germany, across two lunar cycles (N 10 cm bins sampled = 27,054). Estimated effect sizes are shown for fixed effects with 95% confidence intervals and random effects show the variance attributed to date (*N* = 4) and profiles (*N* = 54) as calculated with the bootstrap method (10,000 iterations). Bold text represents significant effects.

Fixed effects	Cladocera	Copepoda
	β (95% CI)	β (95% CI)
Intercept	1.58 (1.38, 1.76)	1.71 (1.64 1.80)
Size class (compared to small)		
*Medium*	-0.03 (-0.06, 0.00)	-0.01 (-0.04, 0.02)
*Large*	**-0.09 (-0.13**,** -0.07)**	-0.01 (-0.04, 0.02)
Light (twilight to moonlight, lx)	**-0.03 (-0.07**,** -0.00)**	-0.02 (-0.05, 0.02)
Temp	**0.10 (0.08**,** 0.13)**	**-0.04 (-0.06**,** -0.02)**
Chl *a*	**0.10 (0.08**,** 0.12)**	**0.17 (0.15**,** 0.20)**
Lx : size class (*medium*)	-0.01 (-0.06, 0.03)	-0.00 (-0.04, 0.05)
Lx : size class (*large*)	0.05 (-0.00, 0.09)	0.05 (-0.00, 0.09)
Temp : size class (*medium*)	**0.19 (0.16**,** 0.22)**	-0.03 (-0.06, 0.02)
Temp : size class (*large*)	**0.43 (0.39**,** 0.46)**	**0.17 (0.14 **,**0.20)**
Lx : Temp	**0.12 (0.08**,** 0.16)**	**0.11 (0.07**,** 0.15)**
Chl *a* : size class (*medium*)	**-0.04 (-0.07**,** -0.01)**	**0.06 (0.02**,** 0.08)**
Chl *a* : size class (*large*)	**-0.08 (-0.11**,** -0.04)**	**0.12 (0.08**,** 0.15)**
Lx : Chl *a*	-0.03 (-0.08, 0.02)	-0.02 (-0.07, 0.02)
Lx : Temp : size class (*medium*)	**-0.08 (-0.13**,** -0.02)**	**-0.09 (-0.14**,** -0.03)**
Lx : Temp : size class (*large*)	**-0.24 (-0.28**,** -0.17)**	**-0.15 (-0.20**,** -0.09)**
Lx : Chl *a* : size class (*medium*)	0.01 (-0.04, 0.09)	-0.07 (-0.13, 0.00)
Lx : Chl *a* : size class (*large*)	**0.07 (0.00**,** 0.13)**	-0.03 (-0.01, 0.04)
**Random effects**	**σ2 (95% CI)**	**σ2 (95% CI)**
Date	0.04 (0.01, 0.10)	0.00 (0.00, 0.02)
Profile	0.02 (0.01, 0.02)	0.01 (0.01, 0.01)

### Copepoda

#### Temperature

In darkness, large copepods were increasingly concentrated in warmer water layers, while medium and small individuals became less abundant under the same conditions (Table 2; Fig. 5a). Under brighter conditions, however, copepods of all sizes were more concentrated in warmer water layers, with small individuals displaying the strongest response (Table 2; Fig. 5b).

#### Food availability

Copepod density increased with in water layers with higher chlorophyll-*a* concentrations, with the strongest response observed in large individuals, followed by medium and small copepods (Table 2; Fig. 5c). Unlike cladocerans, copepods’ response to food availability remained consistent regardless of light conditions at night (Table 2).

## Discussion

Light is the primary proximate factor influencing zooplankton DVM, but historical methodological limitations e.g. by depth-integrated net tow sampling of zooplankton or acoustics have resulted in a gap in high-resolution data capturing fine-scale movements differentiated by taxa and size^1^. This gap has limited our understanding of how variation between functional groups, such as taxonomic and size classes, shapes migration patterns, particularly in response to subtle nighttime light changes, such as those caused by moonlight. To address this, we leveraged recent optical techniques by using the mDPI combined with image-recognition algorithms to capture unprecedented spatiotemporal and size-resolved data on zooplankton vertical movements in situ, especially in relation to subtle nocturnal light variations, such as changes in moonlight intensity. This enabled detailed tracking of dynamic, size-dependent behaviours that would not be detected with traditional net-based or acoustic methods, lacking sufficient taxonomic or spatial resolution. As expected, both cladocerans and copepods migrated into darker waters during the day, with larger individuals exhibiting the strongest migratory response. This is consistent with large zooplanktons higher predation risk and greater swimming capacity, which allows for deeper or more extensive vertical movements^51–54^. However, our findings reveal a more complex relationship between moonlight and zooplankton behaviour. Under full moon conditions, large zooplankton moved to significantly deeper water depths, occupying darker layers, while smaller zooplankton showed more variable vertical positioning, influenced by both light levels and environmental conditions. This indicates that effects of moonlight are size-dependent, reflecting nuanced risk and habitat trade-offs.

Under moonlight, we hypothesized that small individuals would not change vertical position in response to subtle changes in light at night due to an inherently low predation risk, consistent with previous observations of macrozooplankton^55^. Contrary to this, we observed that during full moons, small individuals moved into warmer, brighter surface layers. This upward shift is unlikely to reflect positive phototaxis, as small individuals did not consistently occupy the shallowest, brightest water layers, but instead showed variable depth distributions that were better explained by temperature and food availability. Instead, our results suggest an indirect, light-mediated effect. When larger, more vulnerable individuals avoided illuminated surface layers under moonlight to reduce predation risk, competitive pressure in these warm waters was reduced. This allowed small zooplankton to exploit these favourable conditions due to their lower vulnerability to visual predators.This dynamic redistribution aligns with ecological theories such as Ideal Free Distribution (IFD^56^), Unified Foraging Theory (UFT^57^), and Unequal Competitor models^58^, which predict that organisms adjust their habitat use based on the interplay of risk, resource availability, and competition. In a meso- to eutrophic lake like Stechlin, with patchy resources and a robust planktivorous fish population^32,33,59^ such size-dependent habitat partitioning is likely intensified. While further studies directly measuring predation pressure would strengthen this interpretation, our findings show that moonlight can directly and indirectly restructure zooplankton vertical distributions by modulating inter-size-class competition, revealing fine-scale behavioural plasticity detectable only through high-resolution, in situ tracking.

Our results show that vertical positioning was shaped by taxon- and size-specific resource needs, but these were modulated by ambient light conditions. These factors interacted in ways that were contingent on the presence or absence of nocturnal light. In a lake like Stechlin, where we found a deep chlorophyll-*a* maximum, but also a deep phycoerythrin maximum indicating poor food quality, zooplankton face a trade-off: warmer, food-poor surface waters versus cooler deep layers that contain higher phytoplankton biomass, but with potentially low nutritional quality^33,60,61^. On dark nights, when predation risk is relaxed, large cladocerans were more abundant in the warmer upper layers, likely to balance thermal needs after daytime foraging in cold, deep waters—a behaviour consistent with thermal compensation strategies seen in *Daphnia*^26,62,63^. However, under moonlit conditions, larger cladocerans avoided upper layers altogether, highlighting how light (perceived predation risk) overrides or constrains resource driven movement. In contrast, smaller cladocerans responded more strongly to chlorophyll-*a* concentrations than to temperature, suggesting that their distribution was driven primarily by food requirements, reflecting elevated nutritional demands for growth^64^. Yet even this response was shaped by light: the increased surface occupancy of small individuals under moonlight may reflect reduced competition with light-averse large individuals, rather than a direct response to food availability alone. Copepods, by comparison, showed a stronger and more consistent preference for food-rich zones regardless of light levels, in line with evidence that food availability is the dominant factor for copepod growth and reproduction^65–68^. This was especially evident in large copepods, where resource access is critical for maximizing clutch size^66,67^. Compared to *Daphnia* and other cladocerans, copepods are also known to be more efficient in escaping visual predators such as fish (e.g. Adams et al.^69^) which could also help explain this difference in vertical distribution of copepods and cladocerans. Using mesocosms in Lake Stechlin we have frequently recorded much higher relative content of large cladocerans, especially *Daphnia* spp. compared to copepods in the guts of planktivorous fish, also when the abundance of *Daphnia* and other cladocerans were very low (Nejstgaard et al. unpbl.). In accordance, a multiyear study in Lake Stechlin also show a strong negative correlation between fish abundances and *Daphnia*, while the correlation between fish and copepods were positive^37^. We therefore suggest that copepods may be able to reduce their predation risk compared to cladocerans at similar light conditions. Overall, these findings suggest that the behavioural trade-offs governing DVM are not driven by light or environmental cues in isolation, but by their interaction—modulated by size, taxa, and ecological context.

While simple averages suggest unexpected patterns, for example copepods appearing deeper during new moons than full moons, our fine-scale, size-resolved measurements combined with GLMM analyses better capture the interactions between light and environmental conditions that shape vertical distributions. Nonetheless, several limitations should be acknowledged. Illuminance, while pragmatic and widely used, is weighted toward human visual perception and does not capture spectral shifts associated with lunar elevation or atmospheric scattering^41^, and future studies could incorporate irradiance-based measurements or spectral modelling to further resolve these effects. Similarly, although the mDPI provides unprecedented spatiotemporal and taxonomic resolution, its cost limited our study to a single camera and therefore a single sampling point per night, with multiple units and teams required to capture simultaneous data across sites. Despite these constraints, this dataset represents a substantial improvement over traditional net or acoustic-based approaches, and future research would benefit from extending observations across complete daily cycles between new and full moon phases and comparing multiple lakes to test the generality of our findings.

Our results highlight the importance of understanding the interplay between light and environmental factors shaping zooplankton behaviour. Given the sensitivity of zooplankton to light fluctuations, our findings have broader implications for understanding the impact of unnatural light sources, such as artificial light at night (ALAN), on zooplankton populations. Conducted in a remote, lake under natural darkness^35^, this study provides a vital baseline to isolate moonlight and environmental effects without ALAN’s confounding influence. While ALAN is known to disrupt zooplankton DVM patterns^70–72^, the role of size in these effects remains unexplored. Larger zooplankton, disproportionately affected by light, may face population restructuring under persistent artificial illumination. These effects may be particularly pronounced in shallower freshwater bodies, where limited depth reduces available dark refuge layers for large, light-sensitive individuals. In shallow urban freshwater bodies, limited refuge depth could lead to size-dependent predation, disproportionately removing large, reproductively important individuals. Prolonged exposure to artificial light could act as an evolutionary driver^73^, which may result in an overall smaller size of zooplankton in urban environments. Given zooplanktons keystone role in regulating algae and sustaining higher trophic levels, such shifts could cascade through freshwater food webs, further destabilising already vulnerable ecosystems^74–76^. While our study focused on a single, temperate lake with a deep chlorophyll maximum and planktivorous fish community, the observed patterns may extend to similar systems. Our high-resolution, size-structured approach offers a powerful tool to test these emerging hypotheses, and may be key to forecasting how ongoing light pollution reshapes aquatic ecosystems. Future research combining in situ light manipulations with predator presence will be critical for forecasting how increasing nighttime illumination will reshape aquatic ecosystems in an increasingly lit world.

## Supplementary Information

Below is the link to the electronic supplementary material.

Supplementary Material 1

Supplementary Material 2

Supplementary Material 3

Supplementary Material 4

Supplementary Material 5

Supplementary Material 6

Supplementary Material 7

Supplementary Material 8

Supplementary Material 9

## Data Availability

The data supporting this study are available in the Zenodo repository at the following DOI: 10.5281/zenodo.18184570.

## References

[1] Bandara, K., Varpe, Ø., Wijewardene, L., Tverberg, V. & Eiane, K. Two hundred years of zooplankton vertical migration research. *Biol. Rev.* **96**, 1547–1589 (2021). 33942990 10.1111/brv.12715

[2] Behrenfeld, M. J. et al. Global satellite-observed daily vertical migrations of ocean animals. *Nature* **576**, 257–261 (2019). 31776517 10.1038/s41586-019-1796-9

[3] Gliwicz, M. Z. A lunar cycle in zooplankton. *Ecology* **67**, 883–897 (1986).

[4] Ringelberg, J. & Van Gool, E. On the combined analysis of proximate and ultimate aspects in diel vertical migration (DVM) research. *Hydrobiologia* **491**, 85–90 (2003).

[5] Hobbs, L. et al. A marine zooplankton community vertically structured by light across diel to interannual timescales. *Biol. Lett.* **17** (2021). 10.1098/rsbl.2020.0810 PMC8086989 33622076

[6] Leach, T. H., Williamson, C. E., Theodore, N., Fischer, J. M. & Olson, M. H. The role of ultraviolet radiation in the diel vertical migration of zooplankton: an experimental test of the transparency-regulator hypothesis. *J. Plankton Res.* **37**, 886–896 (2014).

[7] Webster, C. N. et al. Moonlit swimming: vertical distributions of macrozooplankton and nekton during the Polar night. *Polar Biol.* **38**, 75–85 (2015).

[8] Alldredge, A. L. & King, J. M. Effects of moonlight on the vertical migration patterns of demersal zooplankton. *J. Exp. Mar. Bio Ecol.* **44**, 133–156 (1980).

[9] Gliwicz, M. Z. Predation and the evolution of vertical migration in zooplankton. *Nature* **320**, 746–748 (1986).

[10] Vanderploeg, H. A. et al. Hypoxic zones as habitat for zooplankton in lake erie: refuges from predation or exclusion zones? *J. Exp. Mar. Bio Ecol.* **381**, S108–S120 (2009).

[11] Bahlburg, D. et al. Plasticity and seasonality of the vertical migration behaviour of Antarctic Krill using acoustic data from fishing vessels. *R. Soc. Open. Sci.* **10**, (2023). 10.1098/rsos.230520 PMC10523065 37771962

[12] Okkonen, S., Ashjian, C., Campbell, R. G. & Alatalo, P. Krill diel vertical migration: A diagnostic for variability of wind forcing over the Beaufort and Chukchi seas. *Prog Oceanogr.* **181**, 102265 (2020).

[13] Braun, L. M., Brucet, S. & Mehner, T. Top-down and bottom-up effects on zooplankton size distribution in a deep stratified lake. *Aquat. Ecol.* **55**, 527–543 (2021).

[14] Hart, R. C. & Bychek, E. A. Body size in freshwater planktonic crustaceans: an overview of extrinsic determinants and modifying influences of biotic interactions. *Hydrobiologia* **668**, 61–108 (2011).

[15] Murphy, C. A., Pollock, A. M. M., Strecker, A. & Johnson, S. L. Minimal diel vertical migration and consistent zooplankton capturability in low productivity reservoirs, Oregon. *J. Plankton Res.* **45**, 129–143 (2023).

[16] Czerniawski, R. & Krepski, T. Zooplankton size as a factor determining the food selectivity of Roach (Rutilus Rutilus) in water basin outlets. *Water (Switzerland)* **11** (2019).

[17] Ohlhorst, S. L. Diel migration patterns of demersal reef zooplankton. *J. Exp. Mar. Bio Ecol.* **60**, 1–15 (1982).

[18] Jerling, H. L. & Wooldridge, T. H. Lunar influence on distribution of a calanoid copepod in the water column of a shallow, temperate estuary. *Mar. Biol.* **112**, 309–312 (1992).

[19] Ohman, M. D. & Romagnan, J. B. Nonlinear effects of body size and optical Attenuation on diel vertical migration by zooplankton. *Limnol. Oceanogr.* **61**, 765–770 (2016).

[20] Gastauer, S., Nickels, C. F. & Ohman, M. D. Body size- and season-dependent diel vertical migration of mesozooplankton resolved acoustically in the San Diego trough. *Limnol. Oceanogr.* **67**, 300–313 (2022).

[21] Cohen, J. H. & Forward, R. B. Zooplankton diel vertical Migration - A review of proximate control. in *Oceanography and Marine Biology* 89–122 (2009).

[22] McNaught, D. C. & Hasler, A. D. Rate of movement of populations of daphnia in relation to changes in light intensity. *J. Fish. Res. Board. Can.* **21**, 291–318 (1964).

[23] Gillooly, J. F. Effect of body size and temperature on generation time in zooplankton. *J. Plankton Res.* **22**, 241–251 (2000).

[24] Lampert, W. A field study On the dependence of the fecundity of daphnia spec. On food concentration. *Oecologia* **369**, 363–369 (1978). 10.1007/BF00348062 28309923

[25] Zamora-Terol, S. & Saiz, E. Effects of food concentration on egg production and feeding rates of the cyclopoid copepod Oithona Davisae. *Limnol. Oceanogr.* **58**, 376–387 (2013).

[26] Winder, M., Spaak, P. & Mooij, W. M. Trade-offs in daphnia habitat selection. *Ecology* **85**, 2027–2036 (2004).

[27] Beklioglu, M., Gozen, A. G., Yıldırım, F., Zorlu, P. & Onde, S. Impact of food concentration on diel vertical migration behaviour of daphnia pulex under fish predation risk. *Hydrobiologia* **614**, 321–327 (2008).

[28] Dunn, M. et al. Model-informed classification of broadband acoustic backscatter from zooplankton in an in situ mesocosm. **0**, 1–14 (2023).

[29] Sainmont, J. et al. Inter- and intra-specific diurnal habitat selection of zooplankton during the spring bloom observed by video plankton recorder. *Mar. Biol.* **161**, 1931–1941 (2014).

[30] Ohman, M. D. et al. Zooglider: an autonomous vehicle for optical and acoustic sensing of zooplankton. *Limnol. Oceanogr. Methods*. **17**, 69–86 (2019).

[31] Greer, A. et al. Modular shadowgraph imaging for resolving zooplankton distributions in diverse field and mesocosm settings. *Limnol. Oceanogr. Methods*. 67–86. 10.1002/lom3.10657 (2025).

[32] Mehner, T., Wollrab, S., Gonsiorczyk, T. & Nejstgaard, J. Population response of pelagic fishes (ciscoes, Coregonus spp.) to rapidly accelerated eutrophication of an originally oligotrophic deep lake. *Inl Waters*. **13**, 596–613 (2023).

[33] Casper, S. J. *Lake Stechlin: A Temperate Oligotrophic Lake* Vol. 58 (Springer Science & Business Media, 1985).

[34] Wollrab, S. et al. Fifty years of Limnological data on lake Stechlin, a temperate Clearwater lake. *Sci. Data*. **12**, 1–10 (2025). 40533475 10.1038/s41597-025-05319-8 PMC12177041

[35] Jechow, A., Hölker, F., Kolláth, Z., Gessner, M. O. & Kyba, C. C. M. Evaluating the summer night Sky brightness at a research field site on lake Stechlin in Northeastern Germany. *J Quant. Spectrosc. Radiat. Transf* **181** (2015).

[36] Helland, I. P., Freyhof, J., Kasprzak, P. & Mehner, T. Temperature sensitivity of vertical distributions of zooplankton and planktivorous fish in a stratified lake. *Oecologia* **151**, 322–330 (2007). 17024386 10.1007/s00442-006-0541-x

[37] Mehner, T., Padisak, J., Kasprzak, P., Koschel, R. & Krienitz, L. A test of food web hypotheses by exploring time series of fish, zooplankton and phytoplankton in an oligo-mesotrophic lake. *Limnologica* **38**, 179–188 (2008).

[38] Schulz, M., Koschel, R., Reese, C. & Mehner, T. Pelagic trophic transfer efficiency in an oligotrophic, dimictic deep lake (Lake Stechlin, Germany) and its relation to fisheries yield. *Limnologica* **34**, 264–273 (2004).

[39] Mehner, T. & Kasprzak, P. Partial diel vertical migrations in pelagic fish. *J. Anim. Ecol.* **80**, 761–770 (2011). 21366565 10.1111/j.1365-2656.2011.01823.x

[40] Båtnes, A. S., Miljeteig, C., Berge, J., Greenacre, M. & Johnsen, G. Quantifying the light sensitivity of Calanus spp. During the Polar night: potential for orchestrated migrations conducted by ambient light from the sun, moon, or Aurora borealis? *Polar Biol.* **38**, 51–65 (2015).

[41] Śmielak, M. K. Biologically meaningful moonlight measures and their application in ecological research. *Behav. Ecol. Sociobiol.* **77**, 1–14 (2023).

[42] Robinson, F. W. & Tash, J. C. Feeding by Arizona trout (Salmo apache) and brown trout (Salmo trutta) at different light intensities. *Environ. Biol. Fishes*. **4**, 363–368 (1979).

[43] Rader, R. B., Belish, T., Young, M. K. & Rothlisberger, J. The scotopic visual sensitivity of four species of trout: a comparative study. *West. North. Am. Nat.* **67**, 524–537 (2007).

[44] Bochinski, E. et al. Deep Active Learning for In Situ Plankton Classification. in *Pattern Recognition and Information Forensics ICPR 2018* 5–15 (Springer International Publishing, 2019). 10.1007/978-3-030-05792-3_1

[45] Krizhevsky, A., Sutskever, I. & Hinton, G. E. ImageNet classi cation with deep convolutional neural networks. *Adv. Neural Process. Syst. Syst.* 1097–1105. 10.1016/B978-0-12-374105-9.00493-7 (2012).

[46] Kuznetsova, A., Brockhoff, P. B. & Christensen, R. H. B. {lmerTest} package: tests in linear mixed effects models. *J. Stat. Softw.* **82**, 1–26 (2017).

[47] Faraway, J. J. *Extending the Linear Model with R: Generalized Linear, Mixed Effects and Nonparametric Regression Models* (CRC, 2016).

[48] Gelman, A. & Hill, J. *Data Analysis Using Regression and multilevel/hierarchical Models* (Cambridge University Press, 2006).

[49] Cumming, G. & Finch, S. Inference by eye: confidence intervals and how to read pictures of data. *Am. Psychol.* **60**, 170 (2005). 15740449 10.1037/0003-066X.60.2.170

[50] Pilla, R. M. & Williamson, C. E. Multidecadal trends in ultraviolet radiation, temperature, and dissolved oxygen have altered vertical habitat availability for daphnia in temperate lake Giles, USA. *Freshw. Biol.* **68**, 523–533 (2023).

[51] Brooks, J. L., Dodson, S. I. & Predation *Body Size Composition Plankton* **150** (1964). 10.1126/science.150.3692.28 17829740

[52] O’Brien, W. J. The Predator-Prey Interaction of Planktivorous Fish and Zooplankton 67 (1979).

[53] Dodson, S. & Ramcharan, C. Size-specific swimming behavior of daphnia pulex. *J. Plankton Res.* **13**, 1367–1379 (1991).

[54] Wadhwa, N., Andersen, A. & Kiørboe, T. Hydrodynamics and energetics of jumping copepod nauplii and copepodids. *J. Exp. Biol.* **217**, 3085–3094 (2014). 24948628 10.1242/jeb.105676

[55] De Robertis, A. Size-dependent visual predation risk and the timing of vertical migration: an optimization model. *Limnol. Oceanogr.* **47**, 925–933 (2002).

[56] Parker, G. A. & Sutherland, W. J. Ideal free distributions when individuals differ in competitive ability: phenotype-limited ideal free models. *Anim. Behav.* **34**, 1222–1242 (1986).

[57] Mangel, M. & Clark, C. W. Towards a unified foraging theory. *Ecology* **67**, 1127–1138 (1986).

[58] Grand, T. C. & Dill, L. M. Predation risk, unequal competitors and the ideal free distribution. *Evol. Ecol. Res.* **1**, 389–409 (1999).

[59] Tyler, J. A. & Gilliam, J. F. Ideal free distributions of stream fish : A model and test with Minnows, rhinicthys atratulus. *Ecoloy* **76**, 580–592 (1995).

[60] Lampert, W., McCauley, E. & Manly, B. F. J. Trade-offs in the vertical distribution of zooplankton: ideal free distribution with costs? *Proc. R Soc. B Biol. Sci.* **270**, 765–773 (2003). 10.1098/rspb.2002.2291 PMC1691290 12713752

[61] Giling, D. P. et al. Thermocline deepening boosts ecosystem metabolism: evidence from a large-scale lake enclosure experiment simulating a summer storm. *Glob Chang. Biol.* **23**, 1448–1462 (2017). 27664076 10.1111/gcb.13512

[62] Williamson, C. E., Sanders, R. W., Moeller, R. E. & Stutzman, P. L. Utilization of subsurface food resources for zooplankton reproduction: implications for diel vertical migration theory. *Limnol. Oceanogr.* **41**, 224–233 (1996).

[63] Kessler, K. & Lampert, W. Depth distribution of daphnia in response to a deep-water algal maximum: the effect of body size and temperature gradient. *Freshw. Biol.* **49**, 392–401 (2004).

[64] Gliwicz, M. Z. Food thresholds and body size in Cladocerans. *Nature* **343**, 638–640 (1990).

[65] Ban, S. Effect of temperature and food concentration on post-embryonic development, egg production and adult body size of calanoid copepod eurytemora affinis. *J. Plankton Res.* **16**, 721–735 (1994).

[66] Koski, M. & Kuosa, H. The effect of temperature, food concentration and female size on the egg production of the planktonic copepod acartia bifilosa. *J. Plankton Res.* **21**, 1779–1789 (1999).

[67] Williams, T. D. & Jones, M. B. Effects of temperature and food quantity on the reproduction of Tisbe battagliai (Copepoda: Harpacticoida). *J. Exp. Mar. Bio Ecol.* **236**, 273–290 (1999).

[68] Liu, X., Beyrend, D., Dur, G. & Ban, S. Combined effects of temperature and food concentration on growth and reproduction of eodiaptomus japonicus (Copepoda: Calanoida) from lake Biwa (Japan). *Freshw. Biol.* **60**, 2003–2018 (2015).

[69] Adams, J. B., Bollens, S. M. & Bishop, J. G. Predation on the invasive copepod, Pseudodiaptomus forbesi, and native zooplankton in the lower Columbia river: an experimental approach to quantify differences in prey-specific feeding rates. *PLoS One*. **10**, 1–18 (2015). 10.1371/journal.pone.0144095 PMC4664400 26618851

[70] Berge, J. et al. Diel vertical migration of Arctic zooplankton during the Polar night. *Biol. Lett.* **5**, 69–72 (2009). 18948249 10.1098/rsbl.2008.0484 PMC2657746

[71] Ludvigsen, M. et al. Use of an autonomous surface vehicle reveals small-scale diel vertical migrations of zooplankton and susceptibility to light pollution under low solar irradiance. *Sci. Adv* **4** (2018). 10.1126/sciadv.aap9887 PMC5762190 29326985

[72] Moore, M. V., Pierce, S. M., Walsh, H. M., Kvalvik, S. K. & Lim, J. D. Urban light pollution alters the diel vertical migration of Daphnia. *SIL Proceedings* 27 (2000). (1922).

[73] Hopkins, G. R., Gaston, K. J., Visser, M. E., Elgar, M. A. & Jones, T. M. Artificial light at night as a driver of evolution across urban–rural landscapes. *Front. Ecol. Environ.* **16**, 472–479 (2018).

[74] Carpenter, S. R. et al. Trophic cascades, nutrients, and lake productivity: Whole-lake experiments. *Ecol. Monogr.* **71**, 163–186 (2001).

[75] Pace, M. L. et al. Whole-lake carbon-13 additions reveal terrestrial support of aquatic food webs. *Nature* **427**, 240–243 (2004). 14724637 10.1038/nature02227

[76] Hölker, F., Jechow, A., Schroer, S., Tockner, K. & Gessner, M. O. Light pollution of freshwater ecosystems: Principles, ecological impacts and remedies. *Philos Trans. R Soc. B Biol. Sci* **378** (2023). 10.1098/rstb.2022.0360 PMC10613548 37899012

