# Supplementary material for: High-resolution in situ imaging reveals size-specific moonlight responses in zooplankton diel vertical migration
Source: Sci Rep. 2026 Jan 28;16:4086. doi: 10.1038/s41598-026-36105-0 (PMC12855829; doi:10.1038/s41598-026-36105-0)
Supplement: Supplementary file 1 — Supplementary Material 1 [file 41598_2026_36105_MOESM1_ESM.docx]

## Supplementary Information for:

# High-resolution *in situ* imaging reveals size-specific moonlight responses in zooplankton diel vertical migration

Ashton L. Dickerson^1^*, Andreas Jechow^1,2^, Michelle Nößler^1^, Tim Walles^3^, Stella A. Berger^3^, Franz Hölker^1,4^ and Jens C. Nejstgaard^3*^

^1^ Leibniz Institute of Freshwater Ecology and Inland Fisheries (IGB), Department of Community and Ecosystem Ecology, 12587 Berlin, Germany

^2^ Brandenburg University of Applied Sciences, 14770 Brandenburg a.d.H., Germany

^3^ Leibniz Institute of Freshwater Ecology and Inland Fisheries (IGB), Department of Plankton and Microbial Ecology, Zur alten Fischerhütte 2, 16775 Stechlin, Germany

^4^ Freie Universität Berlin, Institute of Biology, 14195 Berlin, Germany

*** Corresponding authors:** Ashton L Dickerson, [ashton.l.dickerson@igb-berlin.de](mailto:ashton.l.dickerson@igb-berlin.de);

Jens C Nejstgaard, [jens.nejstgaard@igb-berlin.de](mailto:jens.nejstgaard@igb-berlin.de)

### Underwater illuminance calculations

We estimated nighttime illuminance in lx (lx) as a measure for underwater “brightness” at each zooplankton concentration bin (10 cm bins to the full depth of 16.7 m). First, illuminance from moonlight at the water surface ($E_{hor,air}$) was obtained via the R package, moonlit (Śmielak, 2023). Second, we calculated net illuminance normal to the angle of incidence of moonlight in air ($E_{0,air}$) by back-calculating using $E_{hor,air}$ and moon elevation ($\propto$):

$$E_{0,air}=\frac{E_{hor,air}}{cos(90-\propto)}$$

We used Snell’s Law to calculate angle of refraction in water ($cos\varphi$) and Fresnel equations to determine reflectance ($R_{total}$), and we derived the following equation to calculate horizontal illuminance immediately below the water surface ($E_{hor,water0m}$).

$$E_{hor,water0m}=E_{0,air}\cdot(1-R_{total})\cdot cos\varphi$$

Next, we calculated the water attenuation coefficient ($k_{d}$) from real-world PAR values obtained by the environmental probes on the LakeLab. We divided the logarithm of PAR at the surface ($E_{hor,water 0 m}$) by the logarithm of PAR at 7 meter depth ($E_{hor,water 7 m}$), yielding:

$${\frac{\ln\frac{E_{hor,water 0 m}}{E_{hor,water 7 m}}}{7}}=k_{d}$$

These values were used to calculate illuminance at each depth (x) to 10 cm bins.

$$E_{hor,x}={E_{hor,water0m}\cdot e}^{-K_{d}x}$$

Values of illuminance below 0.001 lx were treated as zero, representing a conservative estimate of the lowest scotopic visual sensitivity recorded in a fish species (Rader et al., 2007).

**Table S1.** Summary of average zooplankton density (ind*L-1) and size class range across profiles (mean profiles per date = 17, range = 15-19). To split zooplankton into size classes (small, medium, or large), we evenly split the size histogram into three groups for cladocerans and copepods for each profile. This resulted in fairly even classifications across profiles and dates. Zooplankton sizes are reported as the equivalent spherical diameter (ESD) in millimeters (mm), which were calculated by converting pixel-based measurements using a pixel size of 21 micrometers (µm) and then dividing by 1000 to obtain size in mm. This conversion provides an estimate of the zooplankton size based on the image pixel size (21 µm/pixel).

| Date | Abundance, mean ± SD | Size (mm), mean ± SD | | | |
| --- | --- | --- | --- | --- | --- |
|  |  | Small | Medium | Large | Overall |
| *Cladocera* | | | | | |
| 25/08/2022 | 1,266 ± 440 | 0.39 ± 0.04 | 0.53 ± 0.07 | 0.72 ± 0.11 | 0.55 ± 0.15 |
| 13/09/2022 | 952 ± 176 | 0.36 ± 0.03 | 0.45 ± 0.04 | 0.72 ± 0.14 | 0.51 ± 0.17 |
| 29/09/2022 | 645 ± 116 | 0.37 ± 0.03 | 0.47 ± 0.05 | 0.75 ± 0.14 | 0.53 ± 0.19 |
| 12/10/2022 | 436 ± 50 | 0.37 ± 0.03 | 0.45 ± 0.03 | 0.70 ± 0.17 | 0.50 ± 0.17 |
| *Copepod* | | | | | |
| 25/08/2022 | 1,058 ± 108 | 0.36 ± 0.03 | 0.47 ± 0.04 | 0.66 ± 0.11 | 0.49 ± 0.14 |
| 13/09/2022 | 1,053 ± 235 | 0.36 ± 0.03 | 0.45 ± 0.04 | 0.67 ± 0.11 | 0.49 ± 0.15 |
| 29/09/2022 | 991 ± 168 | 0.36 ± 0.03 | 0.46 ± 0.04 | 0.68 ± 0.11 | 0.50 ± 0.15 |
| 12/10/2022 | 759 ± 87 | 0.37 ± 0.03 | 0.46 ± 0.04 | 0.67 ± 0.15 | 0.50 ± 0.15 |


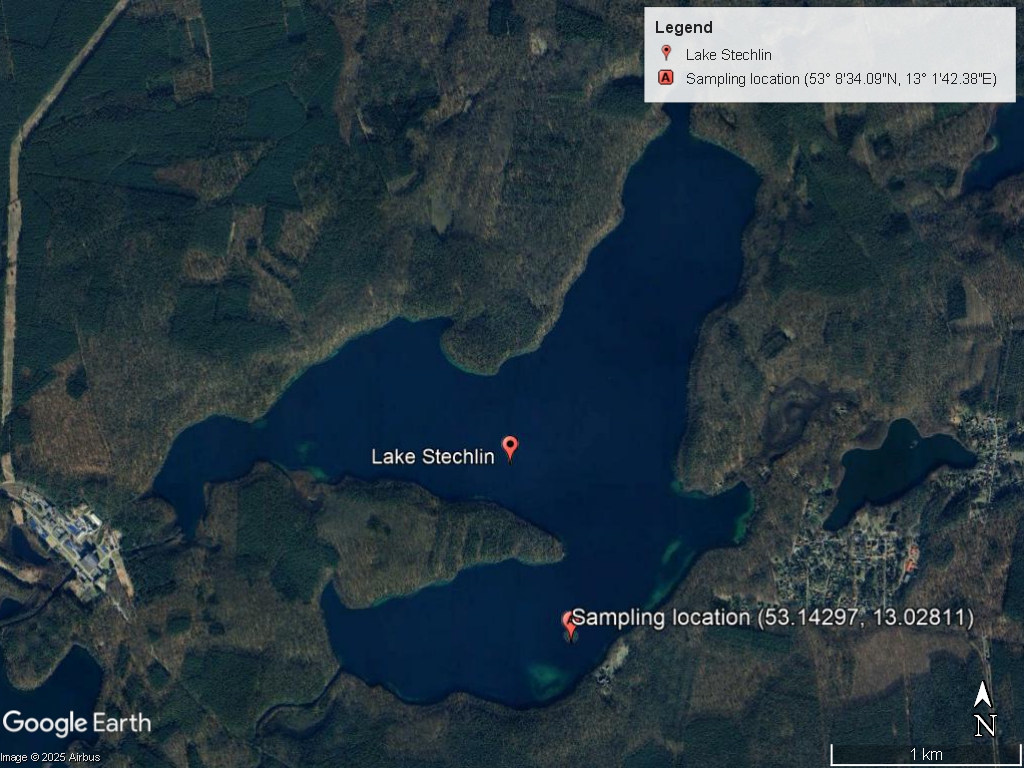


**Figure S1.** Map of Lake Stechlin (53.1430°N, 13.0281°E), showing the sampling location used for all data collection. Depth of point of sampling, 16.7 m. Lake Stechlin is a di-monomictic, meso-eutrophic hardwater lake located in northeastern Germany. It is groundwater-fed, stratified in summer, and well known for its ecological research history and low human impact. Map imagery: Google, © 2025 Airbus.

**Figure S2.** Overview of the modular Deep-focus Plankton Imager (mDPI) including the a) camera housing, b) internal components. Red beams indicate light travelling from the light pod to the camera pod. Pictures show examples of single vignettes (identified objects) of a c) cladoceran and d) copepod


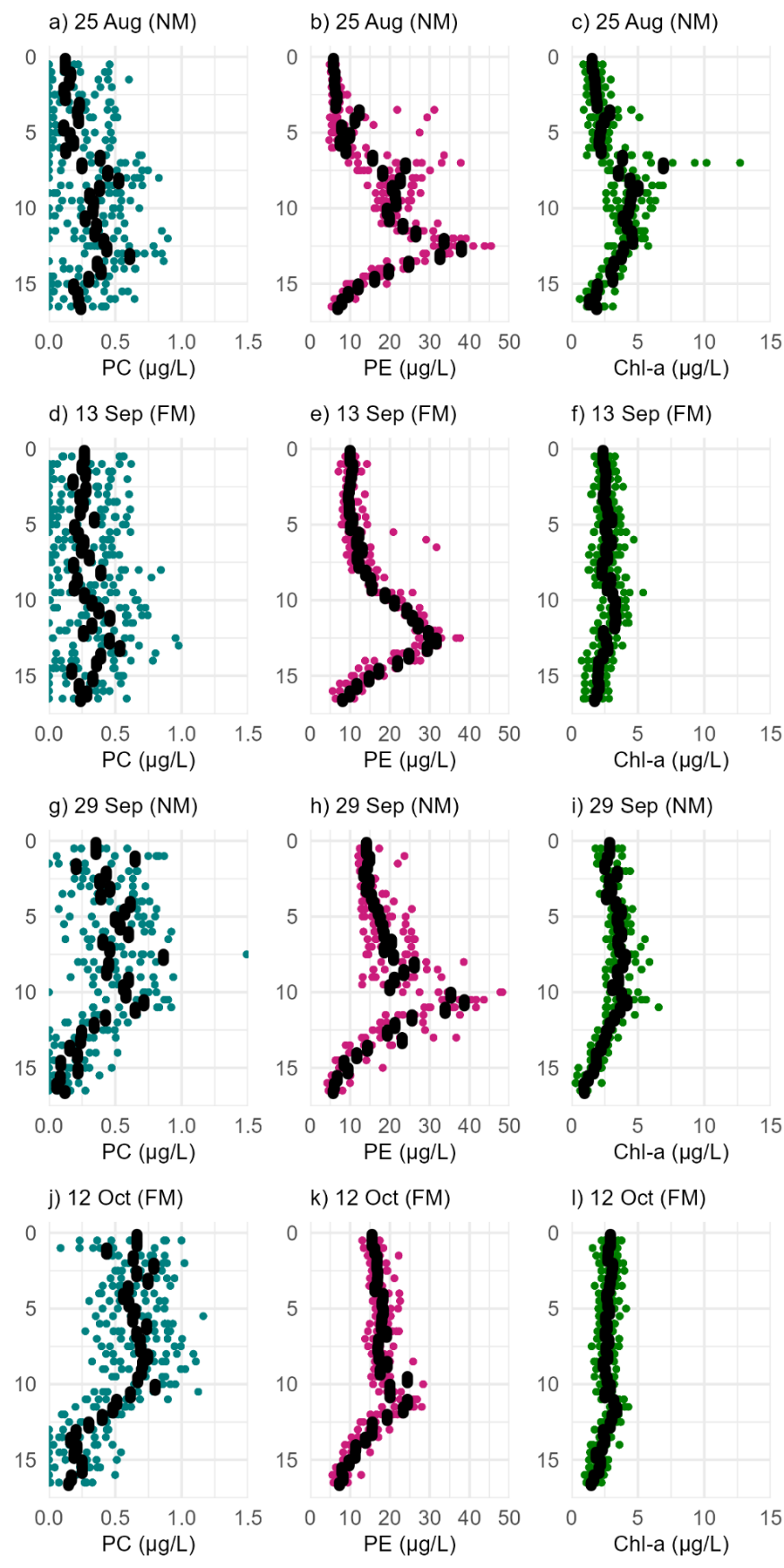


**Figure S3.** Concentrations of **Phycocyanin (PC)**, **Phycoerythrin (PE)**, and **Chlorophyll-*a* (Chl-a)** at different depths for four sampling nights in 2022: (a-c) New moon (25 Aug), (d-f) Full moon (13 Sep), (g-i) New moon (29 Sep), and (j-l) Full moon (12 Oct) at Lake Stechlin, Germany. Average values for each depth are shown as black dots, with individual observations represented by coloured points. Note the scales of concentrations vary for each pigment.
