# Supplementary figures and images for: High-resolution in situ imaging reveals size-specific moonlight responses in zooplankton diel vertical migration
Source: Sci Rep. 2026 Jan 28;16:4086. doi: 10.1038/s41598-026-36105-0 (PMC12855829; doi:10.1038/s41598-026-36105-0)

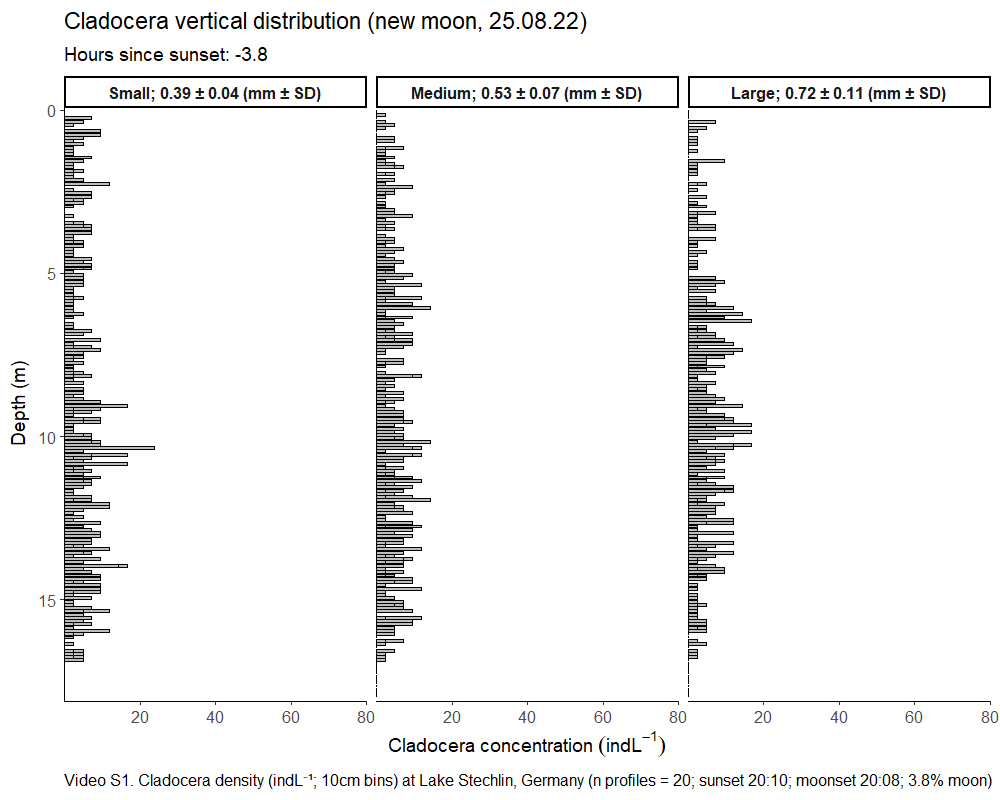

Supplement: Supplementary file 2 — Supplementary Material 2 [file 41598_2026_36105_MOESM2_ESM.gif]

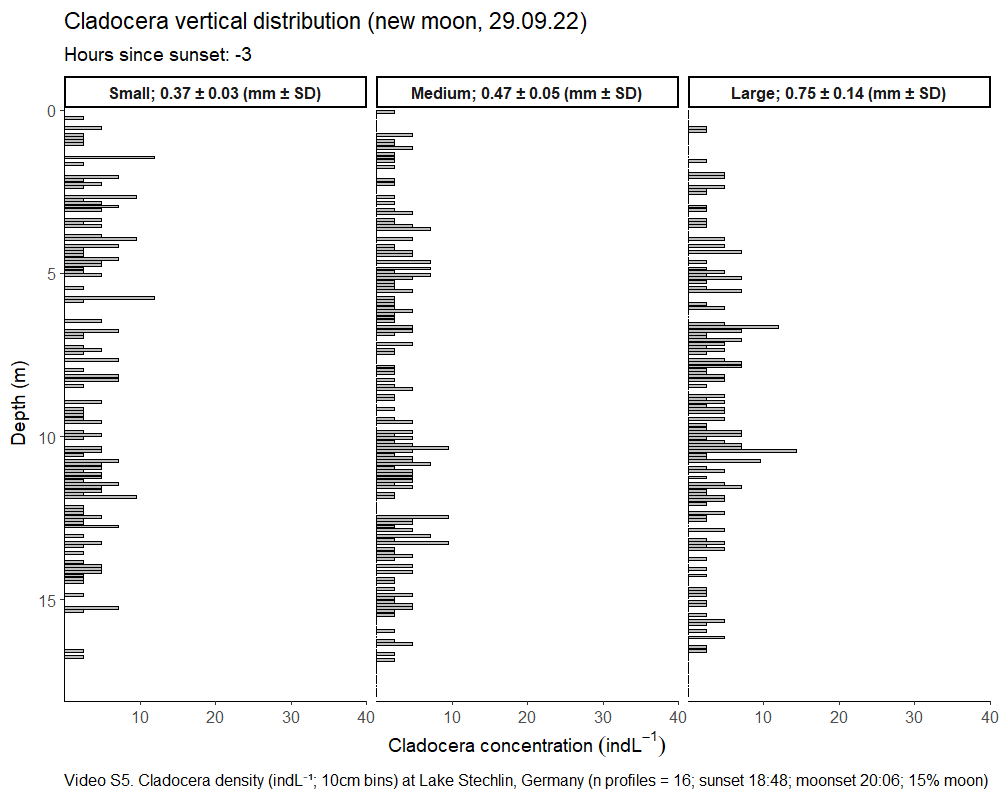

Supplement: Supplementary file 3 — Supplementary Material 3 [file 41598_2026_36105_MOESM3_ESM.gif]

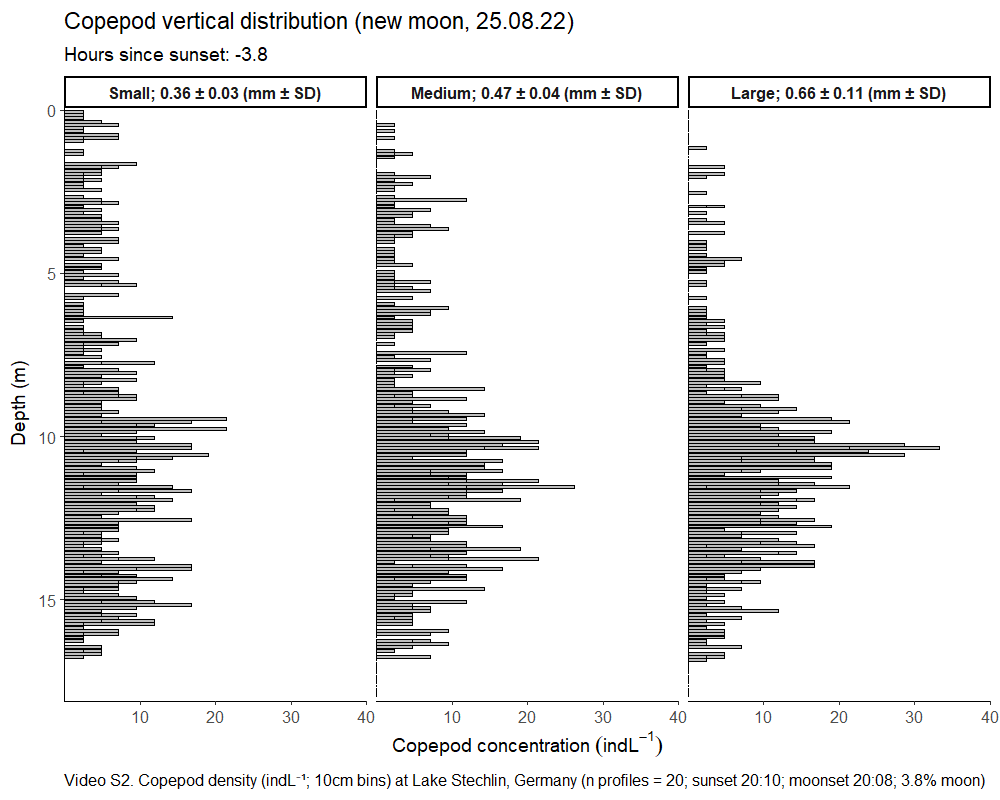

Supplement: Supplementary file 4 — Supplementary Material 4 [file 41598_2026_36105_MOESM4_ESM.gif]

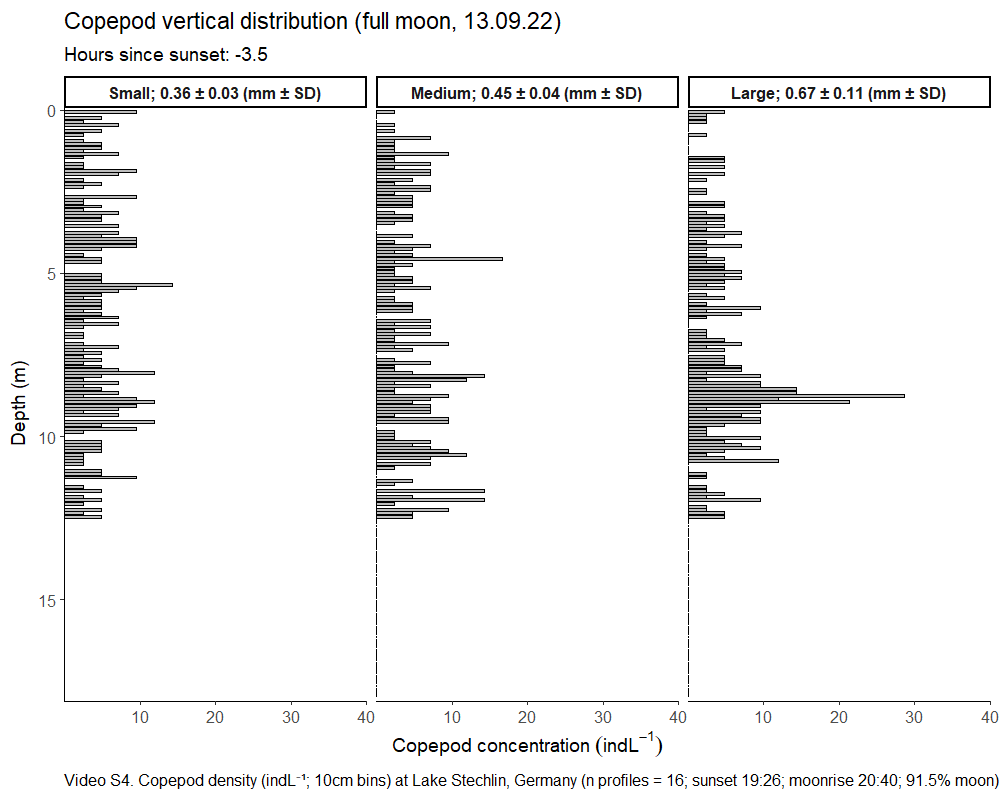

Supplement: Supplementary file 5 — Supplementary Material 5 [file 41598_2026_36105_MOESM5_ESM.gif]

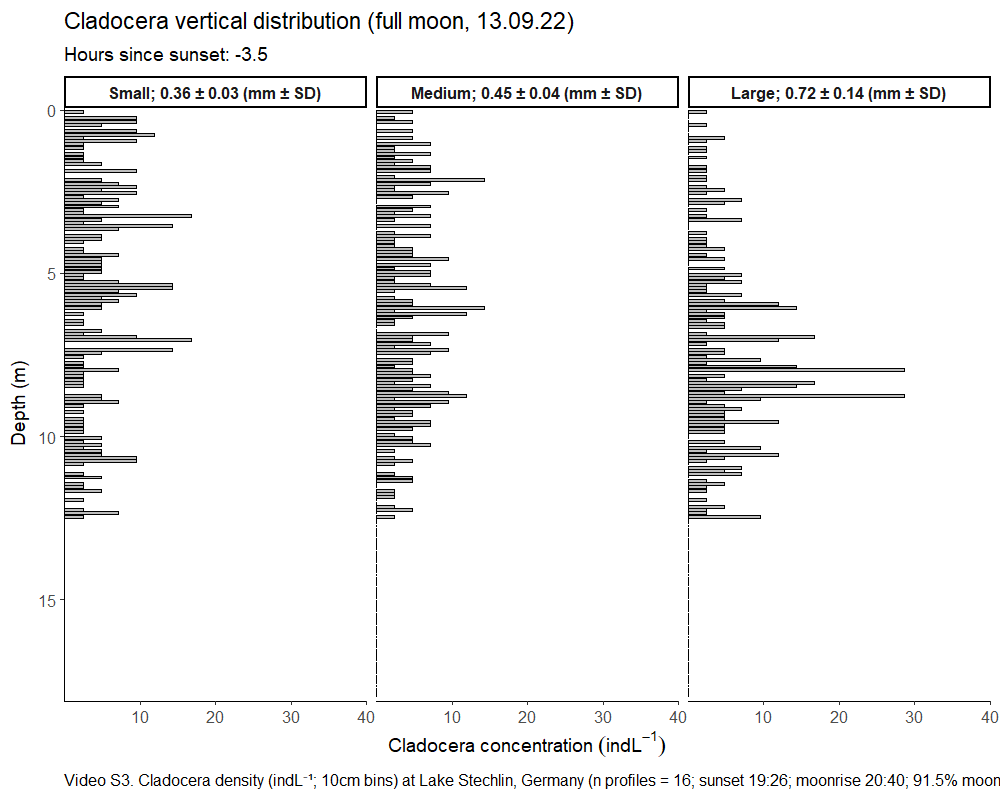

Supplement: Supplementary file 6 — Supplementary Material 6 [file 41598_2026_36105_MOESM6_ESM.gif]

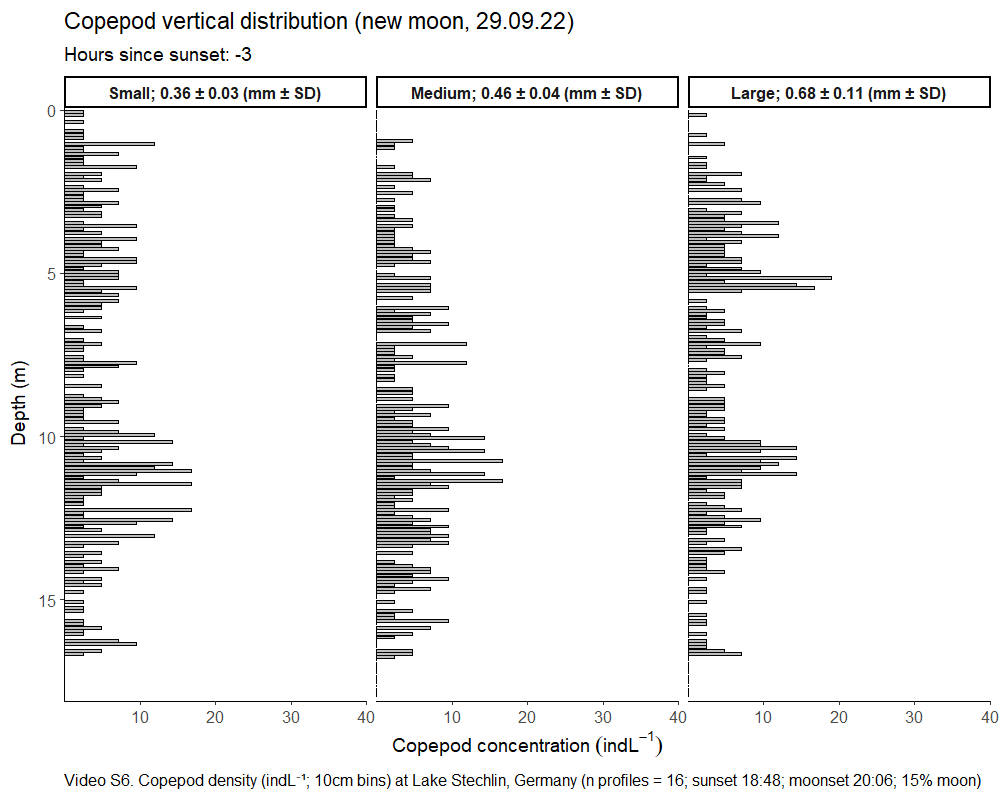

Supplement: Supplementary file 7 — Supplementary Material 7 [file 41598_2026_36105_MOESM7_ESM.gif]

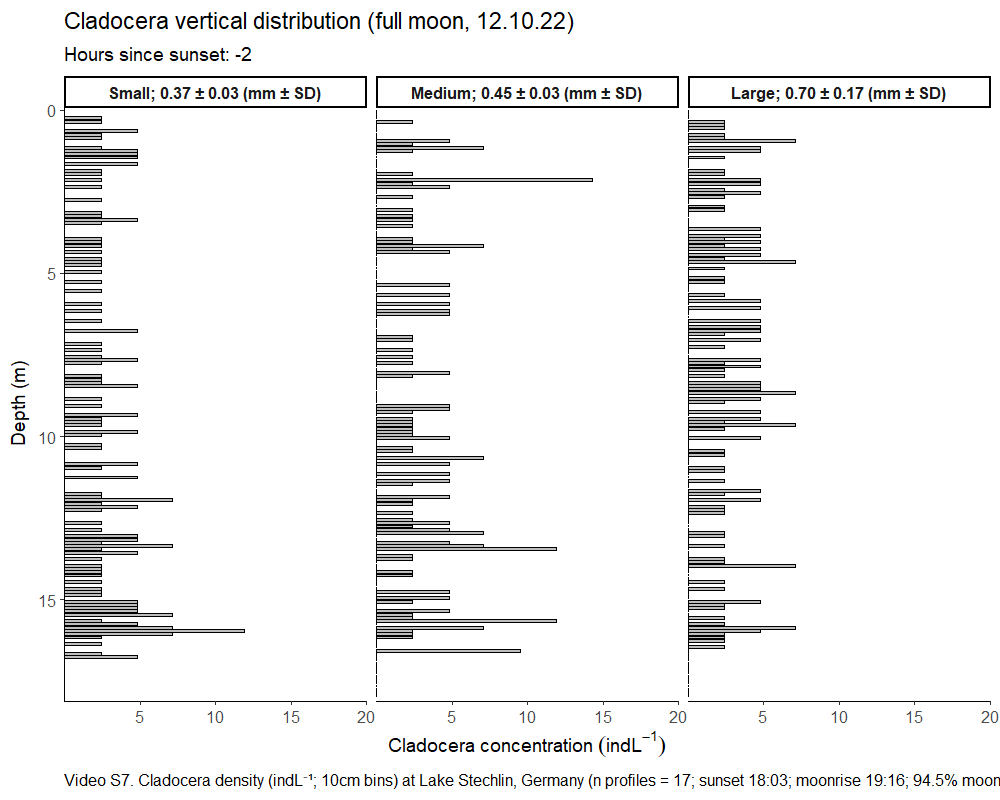

Supplement: Supplementary file 8 — Supplementary Material 8 [file 41598_2026_36105_MOESM8_ESM.gif]

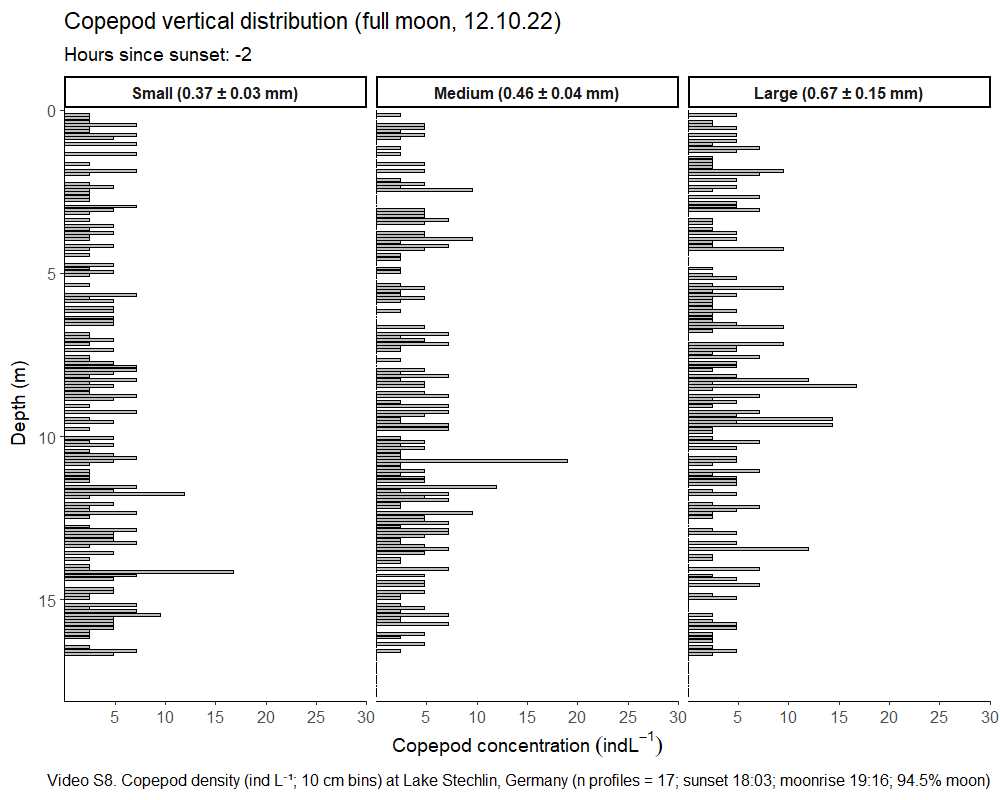

Supplement: Supplementary file 9 — Supplementary Material 9 [file 41598_2026_36105_MOESM9_ESM.gif]
